# Identification and Development of Synovial B-Cell-Related Genes Diagnostic Signature for Rheumatoid Arthritis

**DOI:** 10.1155/2023/9422990

**Published:** 2023-11-25

**Authors:** Jifeng Tang, Jinfang Xia, Huiming Sheng, Jinpiao Lin

**Affiliations:** ^1^Department of Laboratory Medicine, Tongren Hospital, Shanghai Jiao Tong University, School of Medicine, Shanghai, China; ^2^Department of Laboratory Medicine, The First Affiliated Hospital of Fujian Medical University, Fuzhou, China

## Abstract

**Background:**

The aim of the study was to investigate the landscape of B-cell-related gene expression profiling in rheumatoid arthritis (RA) synovium and explore the biological and clinical significance of these genes in RA.

**Methods:**

Expression profiling of synovial biopsies from subjects with 152 RA patients, 22 osteoarthritis (OA) patients, and 28 healthy controls was downloaded from the Gene Expression Omnibus database. Single-sample gene set enrichment analysis (ssGSEA) was performed to evaluate the abundance of infiltrated immune cells, and the results were validated using immunohistochemical staining. GSEA was employed to decipher differences in B-cell-related biological pathways. B-cell-related differential expression genes (BRDEGs) were screened, and BRDEGs-based model was developed by machine learning algorithms and evaluated by an external validation set and clinical RA cohort, then biological functions were further analyzed.

**Results:**

High levels of immune cell infiltration and B-cell-related pathway activation were revealed in RA synovium. BRDEGs were screened, and three key molecular markers consisting of FAS, GPR183, and TFRC were identified. The diagnosis model was established, and these gene markers have good discriminative ability for RA. Molecular pathological evaluation confirmed RA patients with high-risk scores presented higher levels of B-cell activation and RA characteristics. In addition, a competitive endogenous RNA network was established to elucidate the molecular mechanisms of the posttranscriptional network.

**Conclusions:**

We described the B-cell-related molecular landscape of RA synovium and constructed a molecular diagnostic model in RA. The three genes FAS, GPR183, and TFRC may be potential targets for clinical diagnosis and immunoregulatory therapy of RA.

## 1. Introduction

Rheumatoid arthritis (RA) is the most common chronic inflammatory rheumatic disease, characterized by degenerative, progressive, and irreversible intraarticular damage and extra-articular manifestations [1, 2]. A large-scale survey of residents estimated that the prevalence of RA (age-adjusted) in China is 0.28% [3], and the prevalence and incidence of RA in the world are increasing annually [1]. Multiple factors could affect the development of RA [4, 5], and the etiology and pathogenesis of RA remain largely unknown. Synovial microenvironment deterioration in RA development is the major cause of synovial invasiveness and joint integrity loss [6]. Numerous studies have shown that the synovial immune microenvironment is complex, and immune cell infiltration plays a leading role in the synovial microenvironment deterioration [7–9]. Hence, it is of great significance to systematically investigate synovial immune-associative biological alterations, which can help us to gain insights into RA pathogenesis and development of new diagnostic techniques or treatment strategies for RA.

As a chronic autoimmune disease, the disease process of RA spans decades, beginning with the development of several autoantibodies against post-translationally modified proteins [10]. After long-term asymptomatic autoimmunity, tissue tolerance erodes, and joint inflammation ensues as tissue-invasive immune cells emerge. Then, the synovial stromal cells were gradually transited into auto-aggressive effector cells in the context of prolonged inflammation and stromal cell-immune cell interactions, which ultimately led to joint destruction [7]. In this process, B cells, as the autoantibody-secreting cells, have been found to play major roles [8]. In the RA synovial microenvironment, activated autoreactive B cells exert various effector functions that may be relevant to the initiation and maintenance of synovial inflammation [8]. Activated B cells could secrete a large number of inflammatory and regulatory cytokines, formulate tertiary lymphoid structures (follicles), activate autoreactive T cells via antigen presentation and expression of costimulatory molecules, and secrete autoantibodies in RA synovium. The contribution of activated autoreactive B cells to RA eventually leads to the maintenance of disease chronicity [8, 11]. Clinically, therapies targeting B cells, such as anti-CD20 antibody rituximab, have proven effective in RA [12, 13], which also supports the pathogenic role of B cells in RA pathogenesis. Taken together, synovial B cells are important immune mediators in RA synovial microenvironment and play a key role in RA pathologies. Studies on autoreactive B cells in RA synovium would contribute to the elucidation of RA pathogenetic mechanisms and the development of RA diagnostic techniques or therapeutic targets.

RA is currently incurable. Early and timely diagnosis is crucial for the treatment and prognosis of RA patients. The RA clinical diagnosis is mainly based on clinical symptoms, X-ray findings, and classical laboratory indexes [14]. However, the clinical symptoms of RA are diverse. It is easy to miss the diagnosis or have a misdiagnosis in clinical practice, and the search for new diagnostic signatures is highly necessary. Excessive infiltration of B cells as one of the most important characteristics of the ecology of RA synovial microenvironment, B-cell-related signatures may have promising diagnostic potential tools of the RA.

In the present study, we performed a systematic investigation of B-cell-related gene (BRG) signatures in RA synovium using bioinformatics methodology, aiming to explore the potential biological function of the key genes and construct a multigene signature for RA molecular diagnosis. Synovial tissues collected from RA patients were used for validation of the proposed key gene signatures.

## 2. Materials and Methods

### 2.1. Patients

A total of 25 RA patients were recruited from the Shanghai Guanghua Hospital in our study, and all satisfied the 2010 American College of Rheumatology/European League against Rheumatism (ACR/EULAR) classification criteria for RA [15]. In addition, 25 osteoarthritis (OA) patients who met the 2018 ACR guidelines [16] were included as controls. Synovial tissues were obtained from the above patients undergoing routine synovectomy. The detailed clinical information of participants is presented in Table S1. The present study was approved by the Tongren Hospital, Shanghai Jiao Tong University School of Medicine, Shanghai, China (Ethical batch number: AF/SC-11/03.1). The authors declare no violation of the Helsinki Doctrine on human experimentation. Verbal and written informed consent were obtained from all participants.

### 2.2. Immunohistochemical Staining

The immunohistochemical staining for CD20 was performed as below. Briefly, after the removal of synovial tissues at the time of surgery, specimens were immediately washed in cold phosphate-buffered saline (pH 7.4) and then fixed in 4% buffered paraformaldehyde at 4°C for 24 hr. The tissues were dehydrated, embedded in paraffin, and then sectioned sagittally at a thickness of 4 *μ*m. Next, after a series of processes, including dewaxing, rehydration, and antigen repair, the sections were blocked with secondary antibody source serum. After blocking, the sections were incubated with CD20 antibodies (Cell Signalling Technology, Inc., MA, USA; Catalog #: 48750S 1∶200 dilution) overnight. The following day, the sections were incubated with secondary antibodies (Cell Signalling Technology, Catalog #: 8114S) and stained with diaminobenzidine. Digital images were taken by using an Olympus BX53 microscope (Olympus, Tokyo, Japan) and analyzed using Motic DSA software (Motic, Hong Kong, China).

### 2.3. Data Collection and Processing

**Figure 1:**
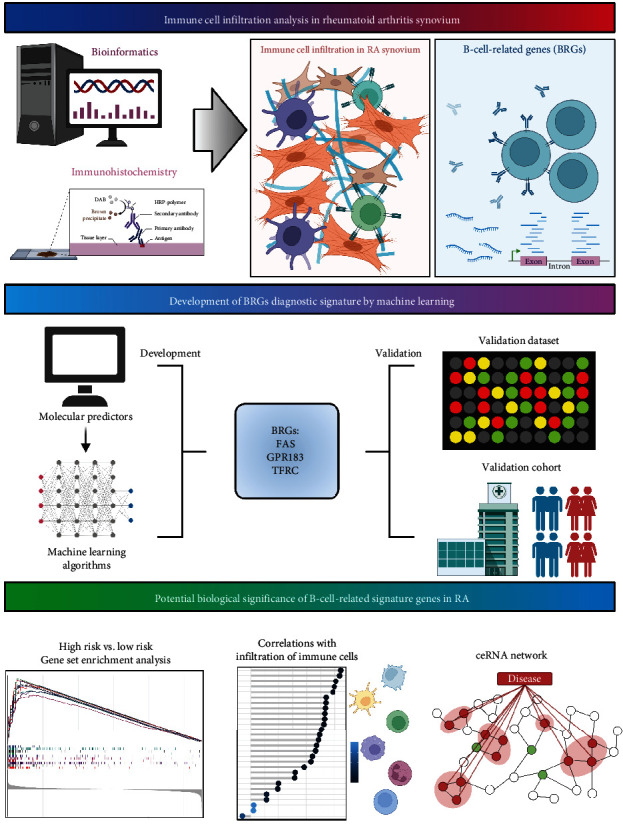
The overall workflow of this study. The overall workflow was created with BioRender.com.

The overall workflow of this study is depicted in Figure 1. In our study, two RA synovial expression profiling by high throughput sequencing, GSE89408 and GSE122616, were recruited from the Gene Expression Omnibus (GEO, http://www.ncbi.nlm.nih.gov/geo/) database. Detailed information of samples and preparation methods are given in the GEO database. Normalization of the RNA read count matrix was performed with the TMM method of the edgeR R package (version 3.42.4). The org.Hs.eg.db R package (version 3.17.0) was applied for mRNA and long noncoding RNA (lncRNA) to ID transform, and the count matrix was transformed into transcripts per kilobase million (TPM) format by executing the count2tpm function of the IOBR R package (version 0.99.9) [17].

### 2.4. Immune Cell Infiltration Analysis

Immune infiltration estimation was performed using the single-sample gene set enrichment analysis (ssGSEA) algorithm by the IOBR R package (version 0.99.9), which is broadly utilized in immune infiltration-related bioinformatics studies [18–21]. The gene panel marking 28 immune cell types used in the algorithm was collected from Charoentong et al. [22] and illustrated in Table S2.

### 2.5. B-Cell-Related Gene Sets Collection

To explore the B-cell-related biological characteristics in RA, we searched for the keyword “B cell” in the Molecular Signatures Database (MSigDB, https://www.gsea-msigdb.org/gsea/msigdb) with C5—gene ontology (GO) as the filtering condition. All returned results were double-checked manually. Finally, 51 B-cell-related pathways and 772 BRGs were screened out for subsequent analysis. The details of BRG sets are provided in Table S3.

### 2.6. Enrichment Analysis

GSEA was performed using clusterProfiler R package [23] (version 4.8.2) to identify markedly aberrant B-cell-related biological alterations in RA. BRG sets were used to conduct GSEA analyses with the GSEA function in clusterProfiler. The gseGO and gseKEGG functions in clusterProfiler were conducted to perform GSEA and further identified the GO and Kyoto Encyclopedia of Genes and Genomes (KEGG) pathways that were significantly enriched in the high- and low-risk groups. In addition, GO and KEGG enrichment analyses were also conducted by clusterProfiler with 55 B-cell-related differentially expressed genes (BRDEGs). All gene sets or pathways with an adjusted *p*-value < 0.05 were considered to be significantly enriched, and the Benjamini and Hochberg method was used for multiple correction.

### 2.7. Protein–Protein Interaction (PPI) Network Analysis

PPI network analysis of the BRDEGs was performed based on the STRING database (https://string-db.org/) with default settings. Cytoscape software (version 3.10.0) was applied to visualize the PPI network according to PPI information. Cytoscape plug-in MCODE (version 2.0.3) was used for searching the significant modules.

### 2.8. Machine Learning Analysis

Nice machine learning algorithms, including XGBoost, logistic regression, LightGBM, RandomForest, AdaBoost, complement naive Bayes (CNB), multilayer perceptron (MLP) neural network, support vector machine (SVM), and K-nearest neighbors (KNN) classifiers were built by python (XGBoost: xgboost version 1.2.1; LightGBM: lightgbm version 3.2.1; others: sklearn version 0.22.1) to construct classification models. Receiver operating characteristic (ROC) curves analysis was performed to screen the most reliable and robust model.

### 2.9. RNA Extraction and Real-Time qPCR

The total RNA of synovial tissue was isolated using the phenol-chloroform method with the TRIzol reagent (Ambion, TX, USA). Then, cDNA was synthesized with the RevertAid First Strand cDNA Synthesis Kit (Thermo Fisher Scientific, MA, USA). Finally, the gene expression was measured by TB Green Premix Ex Taq II kit (Takara Biotechnology, Dalian, China) on Applied Biosystems 7500 with 7500 system software (Applied Biosystems, CA, USA). The sequences of the primers used in the present study were designed using the NCBI Primer-Blast (NCBI Web site) and listed as below: FAS-upstream primer: 5′-CAACAACCATGCTGGGCATC-3′, FAS-downstream primer: 5′-ACCTGGAGGACAGGGCTTAT-3′; GPR183-upstream primer: 5′-TCAATTGCTGCATGGACCCT-3′, GPR183-downstream primer: 5′-CACTGACTTGCCGTTTCAGC-3′; TFRC-upstream primer: 5′-GAACTACACCGACCCTCGTG-3′, TFRC-downstream primer: 5′-GTGCTGTCCAGTTTCTCCGA-3′; GAPDH-upstream primer: 5′-GGAGCGAGATCCCTCCAAAAT-3′, GAPDH-downstream primer: 5′- GGCTGTTGTCATACTTCTCATGG-3′.

### 2.10. Construction of Competitive Endogenous RNA (ceRNA) Networks

Differentially expressed lncRNAs (DElncRNAs) and BRDEGs FAS, GPR183, and TFRC were selected for ceRNA network analysis. Target microRNAs (miRNAs) of DElncRNAs were predicted using the ENCORI online tool (https://rnasysu.com/encori/index.php), while target miRNAs of three BRDEGs were predicted by the miRWalk database (http://mirwalk.umm.uni-heidelberg.de/). Finally, the miRNA–mRNA and lncRNA–miRNA binding pairs were merged into multiple lncRNA–miRNA–mRNA regulatory axes and visualized in Cytoscape software.

### 2.11. Statistical Analysis

All data were processed and plotted using the R software (version 4.3.1). LASSO regression was performed using the glmnet R package (version 4.7-1) to select optimization variables through dimensionality reduction. ROC curve analysis was performed via the pROC R package (version 1.18.4). Comparisons among multiple groups were analyzed using the Kruskal—Wallis rank-sum test, and correlations were calculated by Spearman's correlation. All statistical tests were two-tailed, and *p*-value < 0.05 was considered significant ( ^*∗*^*p* < 0.05,  ^*∗*^ ^*∗*^*p* < 0.01,  ^*∗*^ ^*∗*^ ^*∗*^*p* < 0.001,  ^*∗*^ ^*∗*^ ^*∗*^ ^*∗*^*p* < 0.0001, *ns* = not significant).

## 3. Results

### 3.1. Landscape of Immune Cell Infiltration in RA Synovium

**Figure 2:**
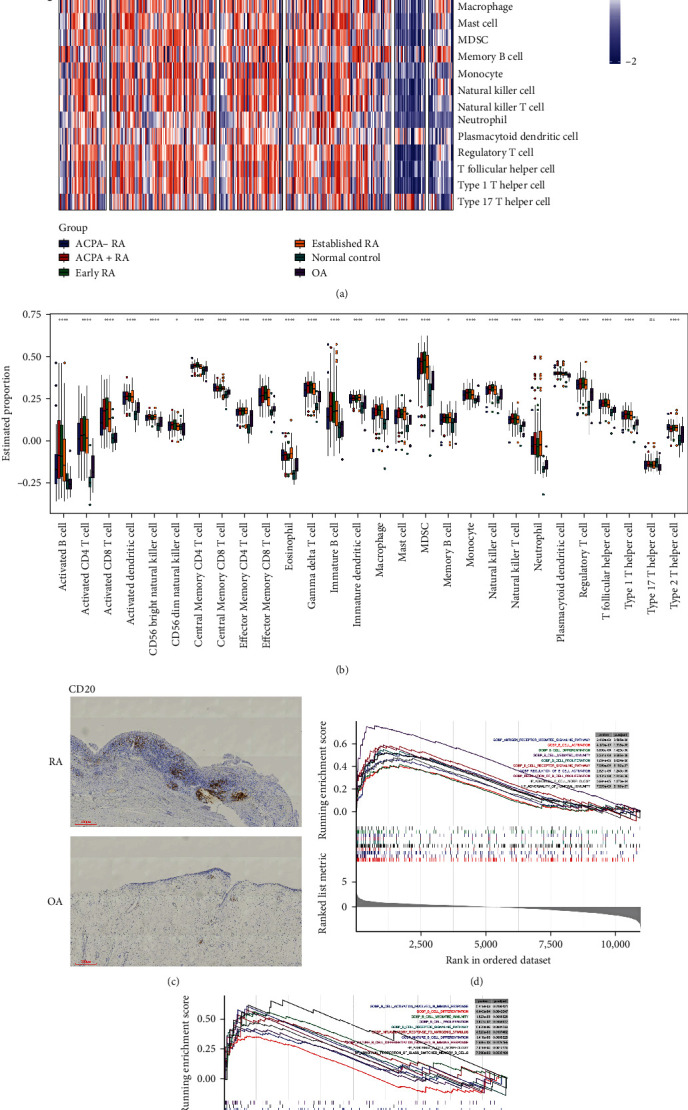
Landscape of immune cell infiltration in RA synovium and aberrant B-cell-related signaling pathways. (a) Heatmap of 28 immune cell infiltration in the synovium of ACPA-positive (+)/-negative (−) RA, early/established RA patients, OA patients, and healthy controls (normal). (b) Boxplot of 28 immune cell infiltration among ACPA-positive (+)/-negative (−) RA, early/established RA patients, OA patients, and healthy controls (normal). (c) Representative images of immunohistochemical staining of CD20 between RA (*n* = 6) and OA synovium (*n* = 6). (d and e) GSEA analysis of 51 B-cell-related biological pathways between RA patients and HC, RA patients, and OA patients.  ^*∗*^*p* < 0.05,  ^*∗*^ ^*∗*^*p* < 0.01,  ^*∗*^ ^*∗*^ ^*∗*^ ^*∗*^*p* < 0.0001, *ns* = not significant.

The GSE89408 dataset contained total RNA sequencing data of joint synovial biopsies from subjects with 152 RA patients, 22 OA patients, and 28 healthy control (HC) was downloaded from the GEO database. Meanwhile, we subgrouped RA patients into anticitrullinated protein antibody (ACPA)-positive/-negative RA, or early/established RA for further analysis according to the clinical characteristics. Based on the ssGSEA method, the relative infiltration of 28 immune cell subpopulations was analyzed in ACPA)-positive/-negative RA, early/established RA, OA, and HC synovium (Figure 2(a)). Unsurprisingly, compared with the other control groups, ACPA-positive/-negative RA, early/established RA synovium was highly infiltrated by various immune cells, including activated B cell, activated T cell, activated dendritic cell, and so on (Figure 2(b)), while the difference of immune cell infiltration among RA subgroups was not apparent, which suggested a microenvironment of excessive immune activation and synovial inflammation in RA.

### 3.2. Screening of BRDEGs

Considered that abnormal activation of B-cell subsets is an important characteristic feature of RA, we further explore the potential significance of B cells in RA synovium. We validated that the number of CD20-positive cells [24] was markedly elevated in RA synovium compared with OA synovium using immunohistochemical analysis (Figure 2(c)). For the sake of finding BRGs, we searched the MSigDB and generated 51 relevant pathways containing 772 BRGs. Then GSEA was performed, and we found B-cell active activation, B-cell differentiation, B-cell-mediated immunity, and other B-cell-related signaling pathways were significantly enriched in both comparison of the RA with OA patient group (RA vs. OA group) and comparison of the RA patients with HCs group (RA vs. HC group), which suggested extensive activation of synovium B cell in RA (Figures 2(d) and 2(e)).

**Figure 3:**
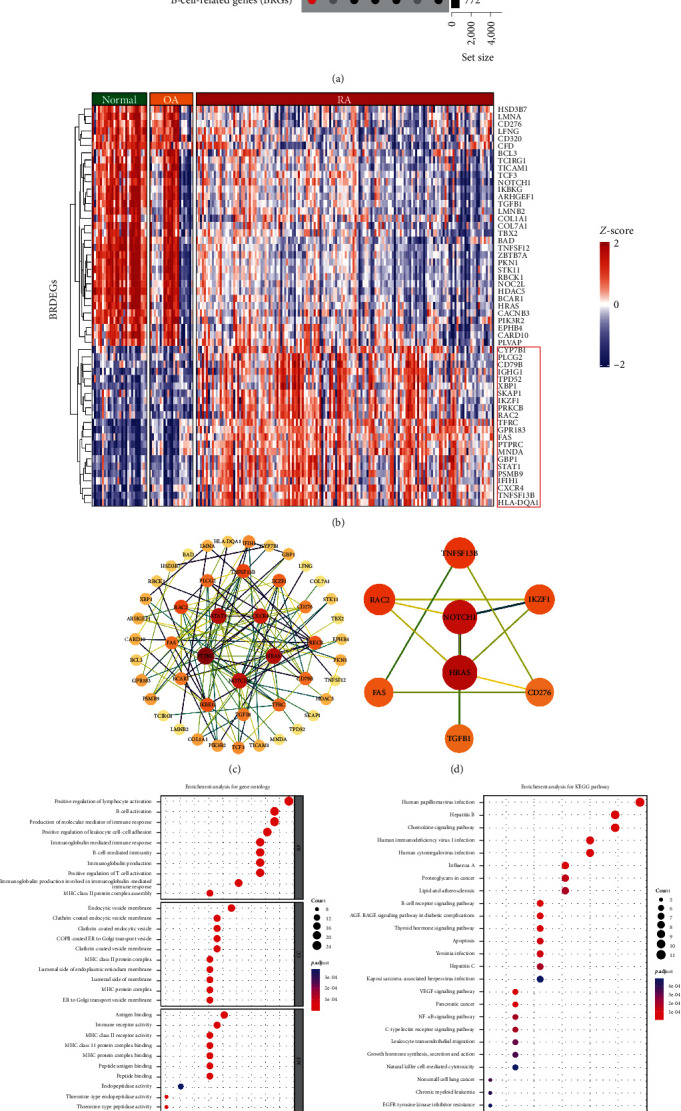
Screening of B-cell-related differential expression genes. (a) UpSet plot of overlap of differential expression genes detected across comparisons among RA patients and HC, RA patients, OA patients, and B-cell-related genes. (b) Heatmap of 55 B-cell-related differential expression genes in RA synovium. (c) Protein–protein interaction network of 55 B-cell-related differential expression genes. (d) The highly interconnected subclusters identified by the Cytoscape plug-in MCODE. (e) GO enrichment analyses of 55 B-cell-related differential expression genes. (f) KEGG enrichment analyses of 55 B-cell-related differential expression genes.

Next, we performed differential gene expression analysis between total RA and two comparison groups, OA and HC, separately with edgeR. With adjusted *p*-value < 0.05 and an absolute value of log2 FoldChange (abs(log2 FC)) > 1 as the cutoff criteria, we identified 3,031 differential expression genes (DEGs) in RA vs. HC group and 1,414 DEGs in RA vs. OA group. Taking the intersection of these DEGs with 722 BRGs, 55 BRDEGs had been screened finally (Figures 3(a) and 3(b)). To determine the interactions between the BRDEGs, we performed a PPI network analysis using the STRING database, which included 48 nodes and 121 edges (Figure 3(c)). Moreover, highly interconnected subclusters consisted 8 nodes and 12 edges from the PPI network were revealed by the Cytoscape plug-in MCODE [25] (Figure 3(d)), which provided potential B-cell-related key targets in RA. In addition, GO and KEGG enrichment analysis indicated that these BRDEGs were mainly enriched in B-cell-related biological processes and signaling pathways, including positive regulation of lymphocyte activation, B-cell activation, immunoglobulin-mediated immune response, as well as relevant pathways such as the B-cell receptor signaling pathway, chemokine signaling pathway, NF-*κ*B signaling pathways (Figures 3(e) and 3(f)). The above results implied that B cells were hyperactivated in RA synovium, and these BRDEGs might be the key targets of RA therapy.

### 3.3. Development of BRGs Diagnostic Signature by Machine Learning

**Figure 4:**
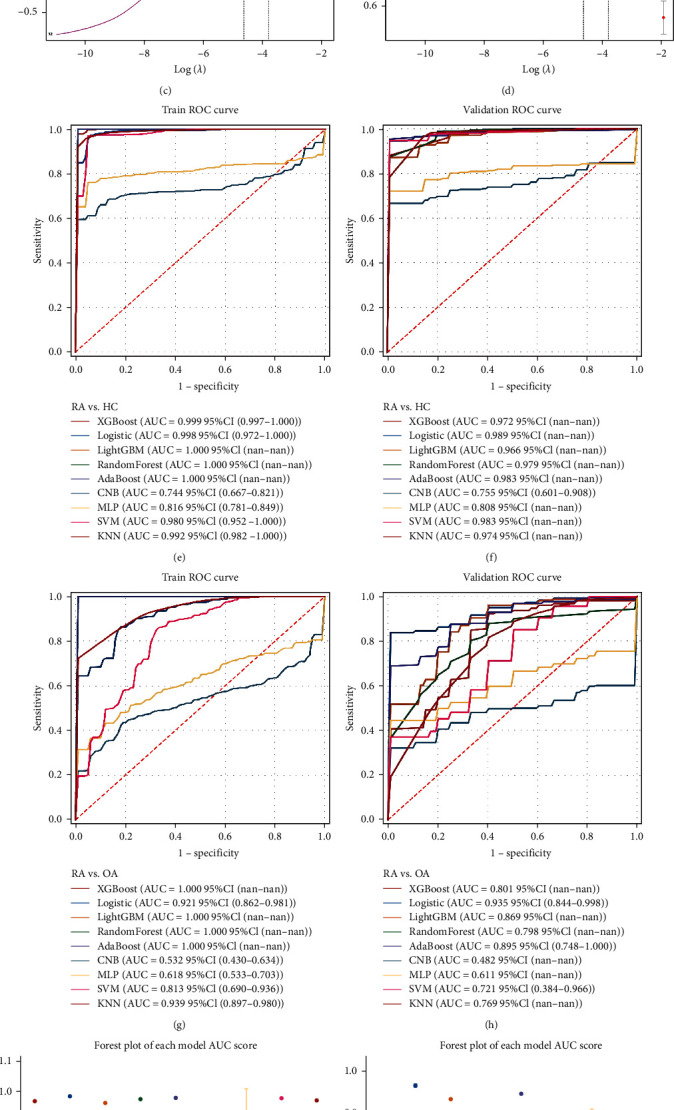
Development of BRGs diagnostic signature by machine learning. (a and c) LASSO coefficient profiles are determined by the optimal lambda. (b and d) Selection of the tuning parameter (lambda) in the LASSO model by the area under the curve (AUC). (e–h) Receiver-operating characteristic curves for nine machine learning models in different comparison groups. (i and j) Forest plot of the AUC score of the nine models in different comparison groups.

To explore the potential diagnostic value of these BRDEGs in RA, 22 upregulated genes were selected (red box in Figure 3(b)) for the final LASSO regression modeling. In RA vs. HC group, the model reached an optimum when lambda was equal to 0.01021, containing five key gene variables, including FAS, GPR183, MNDA, SKAP1, and TFRC (Figures 4(a) and 4(b)). Similarly, the model was optimal when the lambda was 0.00969, and the model contains five key gene variables, including FAS, GPR183, PRKCB, PSMB9, and TFRC (Figures 4(c) and 4(d)) in RA vs. OA group. Finally, three genes, FAS, GPR183, and TFRC, were selected by taking the intersection of two results.

To assess the diagnostic value of three gene variables and select the best-performing model, nine machine learning approaches were conducted with 10-fold-cross validation, including XGBoost, logistic, LightGBM, RandomForest, AdaBoost, CNB, MLP neural network, SVM, and KNN classifiers. The performances of these machine learning algorithms were evaluated using ROC curve analyses, and the results were provided in Tables S4–S7 and Figure 4(e)–4(h). Also, AUC scores across 10-fold cross-validation were calculated, and a forest plot of the mean AUC score with a 95% confidence interval (CI) of the multiple models was shown in Figures 4(i) and 4(j). The above results demonstrated that the logistic regression model performed best and most robust in multiple algorithms. Finally, a risk score using logistic regression was calculated for RA vs. HC group.

### 3.4. Diagnostic Value of B-Cell-Related Genes Diagnostic Signature

**Figure 5:**
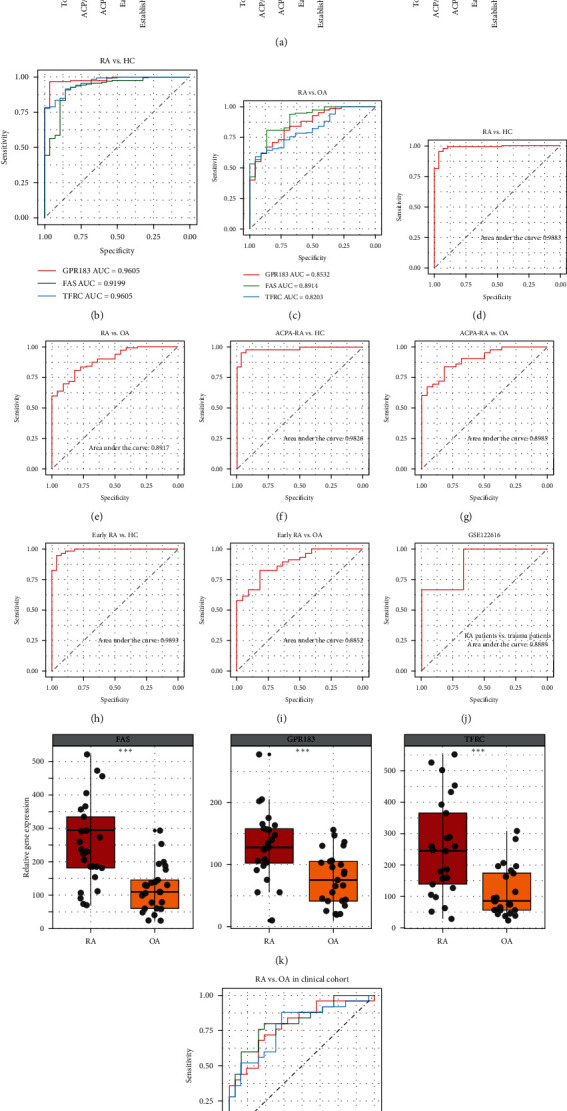
Diagnostic value of B-cell-related genes diagnostic signature. (a) Differential expression of the FAS, GPR183, and TFRC among total RA (RA), ACPA-positive (+)/-negative (−) RA, early/established RA patients, OA patients, and healthy control (HC). (b and c) Receiver-operating characteristic curves (ROC) analysis of FAS, GPR183, and TFRC for RA diagnosis in different comparison groups. (d–i) ROC analysis of the risk score established by FAS, GPR183, and TFRC in different comparison groups. (h) ROC analysis of the risk score established by FAS, GPR183, and TFRC in the GSE122616 dataset. (i) Differential expression of the FAS, GPR183, and TFRC between RA (*n* = 25) and OA (*n* = 25) patients in our clinical cohort. (j) ROC analysis of FAS, GPR183, and TFRC for RA diagnosis in our clinical cohort.  ^*∗*^ ^*∗*^* ^*∗*^p* < 0.001,  ^*∗*^ ^*∗*^ ^*∗*^ ^*∗*^*p* < 0.0001.

Given the difficulty of diagnosis of ACPA-negative RA patients and early RA patients in clinical practice, total RA, ACPA-positive/-negative RA, and early/established RA subgroups were included for subsequent analysis. Relative to the other control groups, the expression levels of FAS, GPR183, and TFRC were significantly higher in total RA and RA subgroups, while no differences between ACPA-positive and -negative RA subgroups as well as early RA and established RA subgroups (Figure 5(a)). This result implied that three genes, FAS, GPR183, and TFRC, did not correlate with ACPA level and disease stage of RA patients. For diagnostic value, the AUCs of FAS, GPR183, and TFRC for RA vs. HC group were 0.9199, 0.9605, and 0.9605, respectively (Figure 5(b)), while the AUCs of FAS, GPR183, and TFRC were 0.8914, 0.8532, and 0.8203 in RA vs. OA group (Figure 5(c)). The performance of the risk score in RA vs. HC group (AUC of 0.9883) was excellent (Figure 5(d)). Even in RA vs. OA group, the AUC of the risk score could reach 0.8917 (Figure 5(e)). Similarly, the performance of the risk score in ACPA-negative RA vs. HC/OA group (AUC of 0.9826 compared with HC and AUC of 0.8985 compared with OA) or early RA vs. HC/OA group (AUC of 0.9893 compared with HC and AUC of 0.8852 compared with OA) are comparable to that in total RA vs. HC/OA group, which further suggest that the roles of these three genes were not associated with the production of ACPA and disease stage (Figure 5(f)–5(i)). Furthermore, we introduced an external validation cohort, GSE122616, to verify the diagnostic power of the risk signature. The AUC of risk score for RA diagnosis was 0.8889, showing a good discriminative power (Figure 5(j)). Moreover, 25 pairs of synovial tissue were obtained from RA and OA patients in the hospital. In our cohort, we found the mRNA levels of FAS, GPR183, and TFRC were increased in RA synovium compared to OA synovium (Figure 5(k)), and the AUCs of these genes for RA diagnosis in our cohort were 0.8144, 0.7904, and 0.7776, respectively (Figure 5(l)), consistent with the aforementioned findings in RNA sequencing results of synovial tissues.

### 3.5. Biological Significance Underlying FAS, GPR183, and TFRC

**Figure 6:**
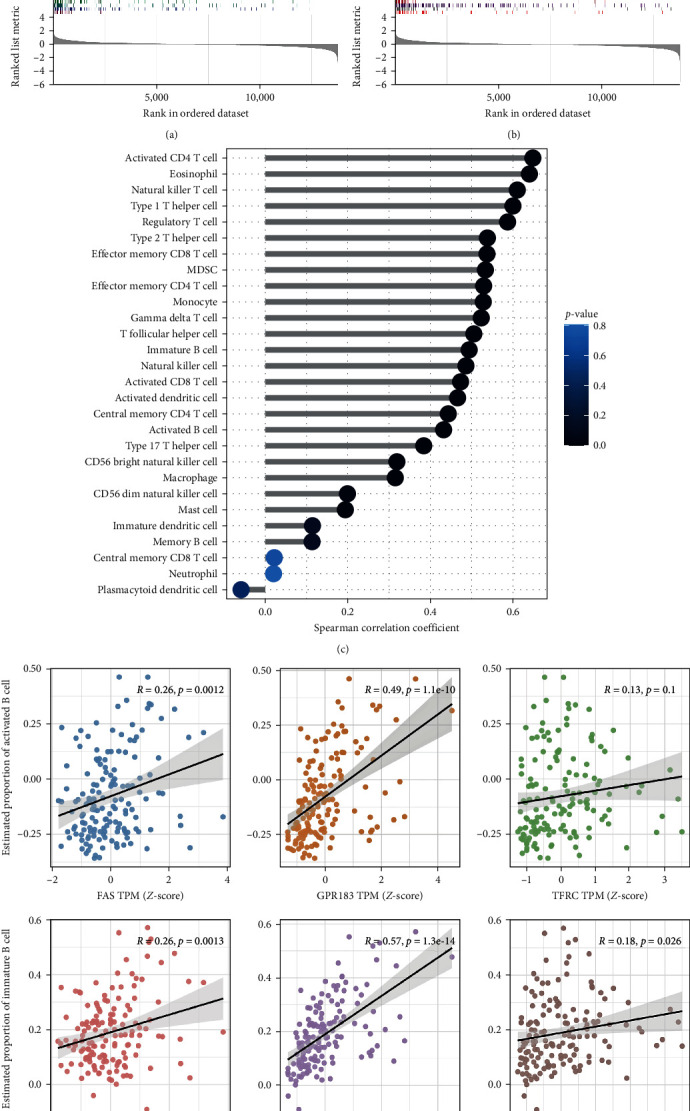
Biological significance underlying FAS, GPR183, and TFRC. (a and b) GSEA for GO and KEGG enrichment between high-risk and low-risk RA groups based on the median risk score established by FAS, GPR183, and TFRC. (c) Correlation between the risk score and immune cell abundance. (d) Correlation between FAS, GPR183, or TFRC with the estimated proportion of activated B cells or immature B cells.

We separated all samples in GSE89408 into high- and low-risk groups based on the median risk score to explore the potential biological function underlying FAS, GPR183, and TFRC. The top 10 for each enrichment results of GSEA for GO between high-risk and low-risk groups revealed that numerous terms of specific functions to B cells such as antigen processing and presentation, immunoglobulin production involved in immunoglobulin-mediated immune response, and MHC class II protein complex were enriched (Figure 6(a)). Meanwhile, the top 10 for each enrichment result of GSEA for KEGG showed immune-related pathways and inflammatory pathways, including antigen processing and presentation, RA as well as NF-*κ*B signaling pathway were significantly enriched in the high-risk group than that in the low-risk group (Figure 6(b)). These findings suggested that the risk score composed of three BRDEGs may be closely related to B-cell activation and production of antibodies, which was one of the best-known characteristics of autoimmune diseases, including RA. Moreover, we further explored the correlation between the risk score and infiltration score of 28 immune cells. As shown in Figure 6(c), the risk score was positively correlated with most immune cells, such as activated CD4 T cells, natural killer T cells, type 1 and 2T helper cells, regulatory T cells, and so on, which implied the ability of the risk score to reflect immune cell infiltration and complex interplay between immune cells in the synovium of RA patients. Also, a correlation between three BRDEGs and activated or immature B-cell infiltration score was further explored. The Spearman's correlation result (Figure 6(d)) demonstrated that GPR183 showed strong correlations with activated B-cell infiltration score (*r* = 0.49, *p* < 0.0001) and immature B-cell infiltration score (*r* = 0.57, *p* < 0.0001), while FAS showed moderate correlations with activated B-cell infiltration score (*r* = 0.26, *p* < 0.01) and immature B-cell infiltration score (*r* = 0.26, *p* < 0.01). However, TFRC showed weak correlations with immature B-cell infiltration score (*r* = 0.26, *p* < 0.05) and no correlation with activated B-cell infiltration score (*r* = 0.13, *p* > 0.05). Taken together, RA patients with high-risk scores may be at high risk of tissue damage and disease progression, more heavily infiltrated by immune cells, and higher inflammatory response in the synovium.

### 3.6. Potential Cerna Network Composed of FAS, GPR183, and TFRC

**Figure 7:**
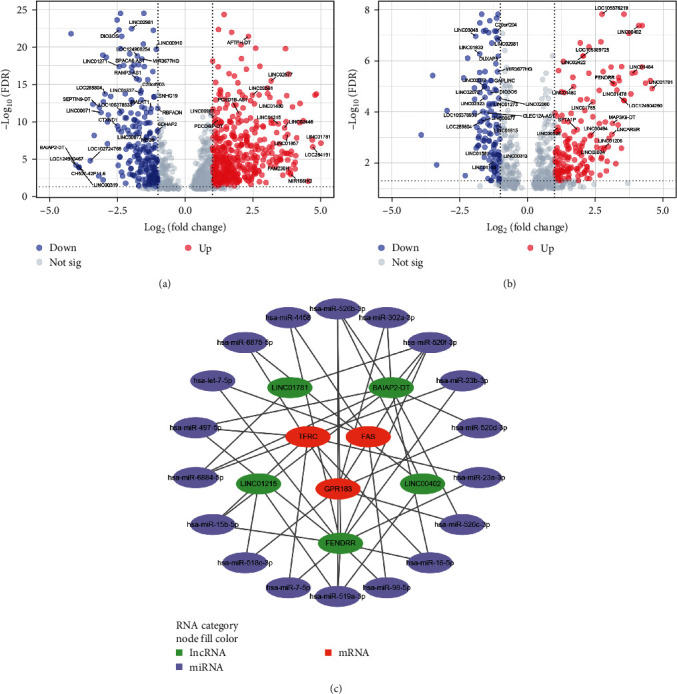
Potential ceRNA network composed of FAS, GPR183, and TFRC. (a and b) Volcano plots of differentially expressed long-chain noncoding RNAs between RA patients and HC, RA patients, and OA patients. (c) CeRNA networks of FAS, GPR183, and TFRC.

We constructed a ceRNA network to reveal the underlying posttranscriptional regulatory mechanisms. In GSE89408, a total of 706 differentially expressed long-chain noncoding RNAs (DElncRNAs) in RA vs. HC group and 341 DElncRNAs in RA vs. OA group were identified (Figures 6(a) and 6(b)). With abs(log2 FC) > 3 as the cutoff criteria, 26 DElncRNAs genes were finally obtained for the subsequent ceRNA network construction by taking the intersection of DElncRNAs between RA vs. HC group and RA vs. OA group. MicroRNAs (miRNAs) interacting with the three BRDEGs and these DElncRNAs were predicted based on the miRWalk database and ENCORI online tool, respectively. After integrating the miRNA–mRNA and lncRNA–miRNA binding pairs, a ceRNA network comprising three BRDEGs, 18 miRNAs, and 5 DElncRNAs was completely developed (Figure 7(c)). In the lncRNA–miRNA–mRNA axis, DElncRNAs may affect three BRDEGs expression through the regulation of miRNAs. Overall, the ceRNA network constructed above provided a potential posttranscriptional regulatory landscape and the selection of non-coding RNA therapeutic targets for the three key BRDEGs.

## 4. Discussion

The pathogenic mechanism underlying RA is complicated, and B cells have been proven to play an essential role in the pathological progression of RA, which is involved in immune activation, inflammation, and production of autoantibodies [7, 8, 11]. Given the core position of B cells in the RA synovial microenvironment, systematic exploration of the BRG expression profile and identification of key genes are particularly warranted for an in-depth understanding of the RA pathogenesis, which is also conducive to advances in molecular diagnosis and immunomodulatory therapy. In this research, we first comprehensively estimated the infiltration of 28 immune cells in RA synovium by ssGSEA, and we found the degree of immune cell infiltration of RA synovium was significantly higher than that of OA and HC synovium. This is to be expected because RA is an autoimmune disease resulted from immunological abnormalities. Meanwhile, we also noticed that infiltration of immune cells such as activated B cell, activated CD4 T cell, activated CD8 T cell, activated dendritic cell, immature B cell in ACPA-positive RA synovium were more highly than those in ACPA-negative RA synovium, which suggested that the degree of uncontrolled autoimmune response of ACPA-positive RA patients is more severe than that of ACPA-negative RA patients. In addition, adaptive immune cell infiltration in ACPA-negative RA synovium was lower than that in ACPA-positive RA synovium but still significantly higher than that in OA and HCs control, which implied that most of patients with ACPA-negative RA may have unknown autoantibodies that were still undiscovered or have delayed appearance of the conventional autoantibodies. In addition, we observed that differences in the immune cell infiltration level in synovium were not evident between early RA and established RA patients compared to OA patients and HC, suggesting immune cell infiltration might emerge before the clinical features of RA develop. Next, considered that the core position of B cells in RA pathophysiology and the production of self-reactive autoantibodies in RA, we explored the BRG expression profile in RA synovium.

Immunohistochemical staining for CD20, a cell surface marker unique to B cells, was performed to confirm B-cell infiltration in RA was higher than that in OA. Also, we demonstrated that B-cell-related signaling pathways were significantly enriched in RA synovium compared to that in OA and HCs control. All the above results have elucidated the necessity of exploring B-cell-associated pathological alterations at the levels of cellular infiltration in RA synovium. Based on the expression profiles of 55 BRDEGs obtained from a series of screening, PPI analysis was performed, and 8 genes, including HRAS, NOTCH1, TNFSF13B, RAC2, IKZF1, FAS, CD276, TGFB1, were identified as B-cell-related key genes, which may be considered as B-cell-related potential therapeutic targets for RA. GO and GEGG analysis revealed that a number of B-cell-related signaling pathways and inflammatory pathways were enriched, such as B-cell activation, immunoglobulin-mediated immune response, B-cell-mediated immunity, B-cell-receptor signaling pathway, NF-*κ*B signaling pathway, and so on. These enrichment analysis results demonstrated that aberrant B-cell activation and inflammatory response were significantly elevated in RA synovium. Overall, we revealed the immune cell infiltration landscape in RA and identified biological functions linked to BRDEGs in RA, providing new insights into the RA pathogenic mechanism of synovial B-cell infiltration and activation.

Using LASSO regression and machine learning algorithms, FAS, GPR183, and TFRC were identified as key BRDEGs in RA. FAS, also known as CD95, is the prototype of a death receptor. Evidence suggests that this receptor not only mediated apoptosis signaling pathway but also mainly implements nonapoptotic signaling pathways such as NF-*κ*B, MAPK, and PI3K that are involved in differentiation, cell migration, survival, and cytokine secretion [26]. In RA, increased concentrations of the soluble Fas/CD95-ligand (sFasL/sCD95L) [27] synovial cells expressed high levels of Fas [28] have been observed in the joints of RA patients. G protein-coupled receptor 183 (GPR183) was discovered in 1993 as an Epstein–Barr virus-induced orphan receptor in Burkitt lymphoma cell lines [29], which is important for rapid and efficient B-cell activation [30]. Many lines of evidence indicate that multiple GPR183 signaling pathways are involved in osteoclast development and the generation of adaptive immune responses, which are central to the development of inflammation in articular spaces. Thus, antagonists of GPR183 signaling may provide significant protection against RA [31–33]. TFRC is the gene-encoded transferrin receptor necessary for cellular iron uptake by the process of receptor-mediated endocytosis [34]. Studies have pointed out that TFRC expression and transferrin receptor synthesis is an early event in B-cell activation [35]. In RA, transferrin receptor expression was largely confined to fibroblasts of the synovial lining layer [36], and serum transferrin receptor levels in RA patients were significantly higher than those in normal groups [37].

Early diagnosis is critical for the RA management. Given molecular signatures can be indicative of biological changes, changes in the biomarker profile detected in synovial fluid might provide promising diagnostic prospects for the early detection of RA [38–40]. The diagnosis model composed of these three genes showed an excellent discriminative ability for distinguishing RA patients from OA patients or HCs. The expression level of these three genes was correlated with the degree of immune cell infiltration in RA synovium. Considered that a fairly high level of immune cell infiltration in RA synovium compared with that in OA and HCs synovium, such a high performance of the model is to be expected. It is worth noting that even within ACPA-negative RA patients and early RA patients, the discriminating ability of this model remained consistent with that in total RA patients, which suggested that these gene signatures may not be involved in ACPA antibody production and disease stage but in B-cell activation. Moreover, the performance of the model was also confirmed by an external validation gene set and synovial tissue obtained from clinical patients in the hospital. In summary, gene signatures, FAS, GPR183, and TFRC, may serve as indicators of an abundance of immune cell infiltration and deleterious molecular pathological alterations in RA patients.

Based on the median risk score composed of FAS, GPR183, and TFRC, RA patients were categorized as low- or high-risk groups. GSEA for GO and KEGG enrichment showed that RA patients in the high-risk group with higher levels of B-cell activation and RA characteristics, which revealed that FAS, GPR183, and TFRC were the hub genes and the abnormal expression of these genes were likely to have important pathological effects in RA development. Also, the ceRNA network was finally constructed to offer potential diagnostic biomarkers and therapy targets for RA.

There are still many limitations to our analysis, and several questions need to be more clearly elucidated. First, the BRDEGs-based risk score required a large-sample validation in clinical practice, and optimal cutoff determination are required before clinical translation. Second, additional studies are needed to clarify the molecular mechanisms of FAS, GPR183, and TFRC in RA development. Lastly, the prediction of the ceRNA regulatory network, including DEGs, DElncRNAs, and predict miRNAs in RA synovium require further validation by in vitro and in vivo experiments.

In conclusion, our study elucidated that the landscape of BRG expression profiles in RA synovium and preliminarily explored the diagnostic value and biological functions of hub BRDEGs, namely, FAS, GPR183, and TFRC, which may be potential targets for clinical diagnosis and immunoregulatory therapy of RA.

## Data Availability

The datasets presented in this study can be found in the GEO database, which is global and public online repositories.

## Supplementary Materials

Supplementary Materials Table S1: the clinical characteristics of participants. Table S2: the gene panel marking 28 immune cell types. Table S3: B-cell-related pathways obtained from Molecular Signatures Database with C5—Gene Ontology. Table S4: the mean result of 10 single runs of the machine learning (ML) model for the training set in RA vs. HC. Table S5: the mean result of 10 single runs of the machine learning (ML) model for the testing set in RA vs. HC. Table S6: the mean result of 10 single runs of the machine learning (ML) model for the training set in RA vs. OA. Table S7: the mean result of 10 single runs of the machine learning (ML) model for the testing set in RA vs. OA.
