# Supplementary material for: Identification and Development of Synovial B-Cell-Related Genes Diagnostic Signature for Rheumatoid Arthritis
Source: J Immunol Res. 2023 Nov 25;2023:9422990. doi: 10.1155/2023/9422990 (PMC10693468; doi:10.1155/2023/9422990)
Supplement: Supplementary Materials — Table S1: the clinical characteristics of participants. Table S2: the gene panel marking 28 immune cell types. Table S3: B-cell-related pathways obtained from Molecular Signatures Database with C5—Gene Ontology. Table S4: the mean result of 10 single runs of the machine learning (ML) model for the training set in RA vs. HC. Table S5: the mean result of 10 single runs of the machine learning (ML) model for the testing set in RA vs. HC. Table S6: the mean result of 10 single runs of the machine learning (ML) model for the training set in RA vs. OA. Table S7: the mean result of 10 single runs of the machine learning (ML) model for the testing set in RA vs. OA. [file 9422990.f1.pdf]

Supplementary Table S1: The clinical characteristics of participants.

Characteristics	Rheumatoid arthritis	Osteoarthritis
Number	25	25
Age (years)[a]	63.25 ± 10.24	65.34 ± 13.25
Sex (Male/Female)	5/20	5/20
Anti-CCP (IU/mL)[b]	102.50 (37.00, 251.50)	/
RF (IU/mL)[b]	67.00 (35.50, 148.50)	/
CRP (mm/h)[b]	10.35 (3.16, 17.78)	/
ESR (mg/mL)[b]	31.00 (24.50, 41.00)	/

[a] Expressed as the median (25th to 75th percentile).

[b] Expressed as the means ± SD (standard deviation).

Abbreviations: anti-CCP, anti-cyclic citrullinated peptide antibody; RF, rheumatoid factor; CRP, C-reactive protein; ESR, erythrocyte sedimentation rate.

Supplementary Table S2: The gene panel marking 28 immune cell types.

Cell types	Genes														
Activated B cell	ADAM28	CD180	CD79B	BLK	CD19	MS4A1	TNFRSF17	IGHM	GNG7	MICAL3	SPIB	HLA-DOB	IGKC	PNOC	FCRL2	BACH2
	CR2	TCL1A	AKNA	ARHGAP25	CCL21	CD27	CD38	CLEC17A	CLEC9A	CLECL1						
Activated CD4 T cell	AIM2	BIRC3	BRIP1	CCL20	CCL4	CCL5	CCNB1	CCR7	DUSP2	ESCO2	ETS1	EXO1	EXOC6	IARS	ITK	KIF11
	KNTC1	NUF2	PRC1	PSAT1	RGS1	RTKN2	SAMSN1	SELL	TRAT1							
Activated CD8 T cell	ADRM1	AHSA1	C1GALT1C1	CCT6B	CD37	CD3D	CD3E	CD3G	CD69	CD8A	CETN3	CSE1L	GEMIN6	GNLY	GPT2	GZMA
	GZMH	GZMK	IL2RB	LCK	MPZL1	NKG7	PIK3IP1	PTRH2	TIMM13	ZAP70						
Central memory CD4 T cell	ABHD3	AHNAK	ANXA2P2	AQP3	ATHL1	BMI1	BZW2	CD63	COL4A1	CYLD	ELMO2	FYN	GLIPR1	GSS	IFITM2	ITGB1
	ITGB2	KLF5	LSP1	NDUFB9	PKM2	SFXN3	SIRPG	SMAD4	STX4	TRADD	VIM	XRCC6				
Central memory CD8 T cell	ACTN4	ADAM12	ADCY9	F13A1	FCER1G	FCGR3B	FGF7	FKBP4	GLUD1	GM2A	GUSB	IL1RN	NOL11	NTRK1	RARA	RNF128
	SIGLEC1	TNFRSF11A	TOX4	UBA52	ULBP1											
Effector memeory CD4 T cell	ATM	CASP3	CASQ1	CD300E	DARS	DOCK9	EXOSC9	EZH2	GDE1	IL34	NCOA4	NEFL	PDGFRL	PTGS1	REPS1	SCG2
	SDPR	SIGLEC14	SIGLEC6	TAL1	TFEC	TIPIN	TPK1	UQCRB	USP9Y	WIPF1	ZCRB1					
Effector memeory CD8 T cell	ACAP1	APOL3	ARHGAP10	ATP10D	C3AR1	CCR5	CD160	CD55	CFLAR	CMKLR1	DAPP1	FCRL6	FLT3LG	GZMM	HAPLN3	HLA-DMB
	HLA-DPA1	HLA-DPB1	IFI16	LIME1	LTK	NFKBIA	SETD7	SIK1	TRIB2							
Gamma delta T cell	ACP5	AQP9	BTN3A2	C1orf54	CARD8	CCL18	CD209	CD33	CD36	CDK5	IL10RB	KLRF1	LGALS1	MAPK7	KLHL7	KRT80
	LAMC1	LCORL	LMNB1	MEIS3P1	MPL	FABP1	FABP5	FADD	MFAP3L	MINPP1	RPS24	RPS7	RPS9	DBNL	CCL13	
Immature B cell	CD22	CYBB	FAM129C	FCRL1	FCRL3	FCRL5	FCRLA	HDAC9	HLA-DQA1	HVCN1	KIAA0226	NCF1	NCF1B	P2RY10	SP100	TXNIP
	STAP1	TAGAP	ZCCHC2													
Memory B cell	AICDA	CCNA2	CDKN3	CLCN5	ENPP1	FCER1A	FCRL4	MYC	RUNX2	SORL1	SOX5	STAT5A	STAT5B	TLR9		
Regulatory T cell	CCL3L1	CD72	CLEC5A	FOXP3	ITGA4	L1CAM	LIPA	LRP1	LRRC42	MARCO	MMP12	MNDA	MRC1	MS4A6A	PELO	PLEK
	PRSS23	PTGIR	ST8SIA4	STAB1												
T follicular helper cell	B3GAT1	CDK5R1	PDCD1	BCL6	CD200	CD83	CD84	FGF2	GPR18	CEBPA	CECR1	CLEC10A	CLEC4A	CSF1R	CTSS	DMN
	DPP4	LRRC32	MC5R	MICA	NCAM1	NCR2	NRP1	PDCD1LG2	PDCD6	PRDX1	RAE1	RAET1E	SIGLEC7	SIGLEC9	TYRO3	CHST12
	CLIC3	IVNS1ABP	KIR2DL2	LGMN												
Type 1 T helper cell	CD70	TBX21	ADAM8	AHCYL2	ALCAM	B3GALNT1	BBS12	BST1	CD151	CD47	CD48	CD52	CD53	CD59	CD6	CD68
	CD7	CD96	CFHR3	CHRM3	CLEC7A	COL23A1	COL4A4	COL5A3	DAB1	DLEU7	DOC2B	EMP1	F12	FURIN	GAB3	GATM
	GFPT2	GPR25	GREM2	HAVCR1	HSD11B1	HUNK	IGF2	RCSD1	RYR1	SAV1	SELE	SELP	SH3KBP1	SIT1	SLC35B3	SIGLEC10
	SKAP1	THUMPD2	TIGIT	ZEB2	ENC1	FAM134B	FBXO30	FCGR2C	STAC	LTC4S	MAN1B1	MDH1	MMD	RGS16	IL12A	P2RX5
	CD97	ITGB4	ICAM3	METRNL	TNFRSF1A	IRF1	HTR2B	CALD1	MOCOS	TRAF3IP2	TLR8	TRAF1	DUSP14			
Type 17 T helper cell	IL17A	IL17RA	C2CD4A	C2CD4B	CA2	CCDC65	CEACAM3	IL17C	IL17F	IL17RC	IL17RE	IL23A	ILDR1	LONRF3	SH2D6	TNIP2
	ABCA1	ABCB1	ADAMTS12	ANK1	ANKRD22	B3GALT2	CAMTA1	CCR9	CD40	GPR44	IFT80					
Type 2 T helper cell	ASB2	CSRP2	DAPK1	DLC1	DNAJC12	DUSP6	GNAI1	LAMP3	NRP2	OSBPL1A	PDE4B	PHLDA1	PLA2G4A	RAB27B	RBMS3	RNF125
	TMPRSS3	GATA3	BIRC5	CDC25C	CDC7	CENPF	CXCR6	DHFR	EVI5	GSTA4	HELLS	IL26	LAIR2			
Activated dendritic cell	ABCD1	C1QC	CAPG	CCL3L3	CD207	CD302	ATP5B	ATP5L	ATP6V1A	BCL2L1	C1QB	SNURF	SPCS3	CCNA1	CEACAM8	NOS2
	SRA1	TNFRSF6B	TREM1	TREML1	RHOA	SLC25A37	TNFSF14	TREML4	VNN2	XPO6	CLEC4C	TNFAIP2	UBD	ACTR3	RAB1A	SLA
	HLA-DQA2	SIGLEC5	SLAMF9													
CD56bright natural killer cell	ABAT	C11orf75	C5orf15	CDHR1	DCAF12	DYNLL1	GPR137B	HCP5	HDGFRP2	KRT86	MLST8	ELMOD3	ENTPD5	FAM119A	FAM179A	CLIC2
	COX7A2L	CREB3L4	CSF1	CSNK2A2	CSTA	CSTB	CTPS	CTSD	FST	GATA2	GMPR	HDC	HEY1	HOXA1	HS2ST1	HS3ST1
	BCL11B	CDH3	MYL6B	NAA16	ClQA	ClQB	CYP27B1	EIF3M								
CD56dim natural killer cell	CYP27A1	DDX55	DYRK2	RPL37A	NOTCH3	AKR7A3	GPRC5C	GRIN1	HLA-E	PORCN	PSMC4	UPP1	IL21R	KIR2DS1	KIR2DS2	KIR2DS5
Eosinophil	GIPR	KRT18P50	LRMP	FOSB	RRP12	GPR183	NR4A3	ST3GAL6	DEPDC5	PDE6C	PKD2L2	GPR65	IL5RA	P2RY14	DACH1	DAPK2
	EMR3															
Immature dendritic cell	ACADM	AHCYL1	ALDH1A2	ALDH3A2	ALDH9A1	ALOX15	AMT	ARL1	ATIC	ATP5A1	CAPZA1	LILRA5	RDX	RRAGD	TACSTD2	INPP5F
	RAB38	PLAU	CSF3R	SLC18A2	AMPD2	CLTB	C1orf162									
Macrophage	AIF1	CCL1	CCL14	CCL23	CCL26	CD300LB	CNR1	CNR2	EIF1	EIF4A1	FPR1	FPR2	FRAT2	GPR27	GPR77	RNASE2
	MS4A2	BASP1	IGSF6	HK3	VNN1	FES	NPL	FZD2	FAM198B	HNMT	SLC15A3	CD4	TXNDC3	FRMD4A	CRYBB1	HRH1
	WNT5B															
Mast cell	ADAMTS3	CPA3	CMA1	CTSG	ARHGAP15	CPM	FCN1	FTL	HSPA6	ITGA9	RNASE3	S100A4	SIGLEC8	SLC6A4	PTGS2	EGR3
	PILRA															
MDSC	CCR2	CD14	CD2	CD86	CXCR4	FCGR2A	FCGR2B	FCGR3A	FERMT3	GPSM3	IL18BP	IL4R	ITGAL	ITGAM	PARVG	PSAP
	PTGER2	PTGES2	S100A8	S100A9												
Monocyte	ASGR2	CFP	ASGR1	CD1D	UPK3A	ACTG1	ANXA5	ATP6V1B2	CFL1	DAZAP2	CTBS	EMR4P	HIVEP2	MARCKSL1	MBP	MMP15
	PNPLA6	TMBIM6	PQBP1	TEX264	IKZF1											
Natural killer cell	AKT3	AXL	BST2	CDH2	CRTAM	CSF2RA	CTSZ	CXCL1	CYTH1	DAXX	DGKH	DLL4	DPYD	ERBB3	F11R	FAM27A
	FAM49A	FASLG	FCGR1A	FN1	FSTL1	FUCA1	GBP3	GLS2	GRB2	LST1	BCL2	CDC5L	FGF18	FUT5	FZR1	GAGE2
	IGFBP5	KANK2	LDB3													
Natural killer T cell	BTN2A2	CD101	CD109	CNPY3	CNPY4	CREB1	CRTC2	CRTC3	CSF2	KLRC1	FUT4	ICAM2	IL32	LAMP2	LILRB5	KLRG1
	HSPA4	HSPB6	ISM2	ITIH2	KDM4C	KIR2DS4	KIRREL3	SDCBP	NFATC2IP	MICB	KIR2DL1	KIR2DL3	KIR3DL1	KIR3DL2	NCR1	FOSL1
	TSLP	SLC7A7	SPP1	TREM2	UBASH3A	YBX2	CCDC88A	CLEC1A	THBD	PDPN	VCAM1	EMR1				
Neutrophil	CREB5	CDA	CHST15	S100A12	APOBEC3A	CASP5	MMP25	HAL	C1orf183	FFAR2	MAK	CXCR1	STEAP4	MGAM	BTNL8	CXCR2
	TNFRSF10C	VNN3														
Plasmacytoid dendritic cell	CBX6	DAB2	DDX17	HIGD1A	IDH3A	IL3RA	MAGED1	NUCB2	OFD1	OGT	PDIA4	SERTAD2	SIRPA	TMED2	ENG	FCAR
	IGF1	ITGA2B	GABARAP	GPX1	KRT23	PROK2	RALB	RETNLB	RNF141	SEC14L1	SEPX1	EMP3	CD300LF	ABTB1	KLHL21	PHRF1

Supplementary Table S3: B-cell related pathways obtained from Molecular Signatures Database with C5–Gene Ontology.

B cell-related signaling pathways	Genes														
GOBP B 1 B CELL DIFFERENTIATION	CD19	CMTM7	MALT1	PLCL2	SLAMF8										
GOBP ANTIGEN RECEPTOR MEDIATED SIGNALING PATHWAY	ABL1	ADA	BANK1	BAX	BCAR1	BCL10	BCL2	BLK	BLNK	BMX	BRAF	BTK	BTN1A1	BTN2A1	BTN2A2
	BTN2A3P	BTN3A1	BTN3A2	BTN3A3	BTNL10P	BTNL2	BTNL3	BTNL8	BTNL9	CACNB3	CACNB4	CARD11	CBLB	CCR7	CD160
	CD19	CD22	CD226	CD247	CD276	CD28	CD300A	CD38	CD3D	CD3E	CD3G	CD79A	CD79B	CD81	CD8A
	CD8B	CEACAM1	CMTM3	CRKL	CSK	CTLA4	DENND1B	DGKZ	DUSP22	DUSP3	EIF2B1	EIF2B2	EIF2B3	EIF2B4	EIF2B5
	ELF1	ERMAP	EZR	FCGR2B	FCHO1	FCRL3	FOXP1	FOXP3	FYB1	FYB2	FYN	GATA3	GBP1	GCSAM	GCSAML
	GPS2	GRB2	HHLA2	HLA-A	HLA-DPB1	HLA-DQB1	HLA-DRB1	HLA-DRB3	HRAS	ICOSLG	IGHA1	IGHA2	IGHD	IGHE	IGHG1
	IGHG2	IGHG3	IGHG4	IGHM	IGKC	IGLC1	IGLC3	IGLC6	IGLC7	IKBKB	IKBKG	INPP5D	ITK	KCNN4	KHDRBS1
	KLHL6	LAPTM5	LAT2	LAX1	LCK	LCP2	LGALS3	LILRB4	LIME1	LPXN	LYN	MALT1	MAP3K7	MAPK1	MEF2C
	MIR18A	MIR19A	MIR34A	MNDA	MOG	MS4A1	NCKAP1L	NECTIN2	NFAM1	NFATC2	NFKB1	NFKBIA	NFKBID	NFKBIZ	PAWR
	PDE4B	PDE4D	PHPT1	PIK3CA	PIK3CD	PLCG1	PLCG2	PLCL2	PLEKHA1	PRAM1	PRKCB	PRKCH	PRKD2	PRNP	PSEN1
	PTPN2	PTPN22	PTPN6	PTPRC	PTPRJ	PVRIG	RAB29	RBCK1	RC3H1	RC3H2	RELA	RFTN1	RIPK2	RNF31	RPS3
	SH2B2	SH2D1A	SHB	SKAP1	SLA2	SLC39A10	SOS1	SPG21	SPPL3	STAP1	STK11	STOML2	SYK	TEC	TESPA1
	THEMIS	THEMIS2	THY1	TMIGD2	TNFRSF21	TRAC	TRAF6	TRAT1	TRBC1	TRBC2	TRDC	TRGC1	TRGC2	TXK	UBASH3A
	UBE2N	VAV3	VTCN1	WNK1	ZAP70	ZC3H12A									
GOBP B CELL ACTIVATION	ABL1	ADA	ADAM17	ADGRG3	AHR	AICDA	AKAP17A	AKIRIN2	APLF	AQP8	ATAD5	ATM	BAD	BAK1	BANK1
	BATF	BAX	BCL2	BCL3	BCL6	BLNK	BMI1	BST1	BST2	BTK	C17orf99	CARD11	CASP3	CASP8	CCR6
	CD180	CD19	CD22	CD27	CD28	CD300A	CD320	CD38	CD40	CD40LG	CD70	CD74	CD79A	CD79B	CD81
	CD86	CDH17	CDKN1A	CEBPG	CHRNA4	CHRNB2	CLCF1	CMTM7	CR1	CR2	CTLA4	CTPS1	CXCR5	DCAF1	DCLRE1C
	DLL1	DNAJB9	DOCK10	DOCK11	EP300	EPHB2	ERCC1	EXO1	EXOSC3	EXOSC6	EZH2	FCGR2B	FCRL1	FCRL3	FLT3
	FNIP1	FOXJ1	FOXP3	FZD9	GAPT	GPR183	GPS2	HDAC4	HDAC5	HDAC9	HHEX	HMCES	HMGB3	HSPD1	ICOSLG
	ID2	IFNA1	IFNA10	IFNA13	IFNA14	IFNA16	IFNA17	IFNA2	IFNA21	IFNA4	IFNA5	IFNA6	IFNA7	IFNA8	IFNB1
	IFNE	IFNK	IFNW1	IGBP1	IKZF3	IL10	IL11	IL13	IL2	IL21	IL27RA	IL2RG	IL4	IL4I1	IL5
	IL6	IL7	IL7R	IL9	INHA	INHBA	INPP5D	IRF2BP2	IRF8	IRS2	ITFG2	ITGA4	ITGB1	ITM2A	JAK3
	KIT	KLF6	KMT5B	KMT5C	LAPTM5	LAT2	LAX1	LEF1	LFNG	LGALS1	LIG4	LRRC8A	LYL1	LYN	MAD2L2
	MALT1	MEF2C	MFNG	MIF	MIR17HG	MIR185	MIR19A	MLH1	MMP14	MNDA	MS4A1	MSH2	MSH6	MZB1	NBN
	NCKAP1L	NDFIP1	NFAM1	NFATC2	NFKBIZ	NHEJ1	NKX2-3	NOD2	NOTCH2	NSD2	NTRK1	ONECUT1	PARP3	PAWR	PAX5
	PRKCB	PRKCD	PRKDC	PRLR	PTK2B	PTPN2	PTPN6	PTPRC	PTPRJ	RABL3	RAG1	RAG2	RASGRP1	RBPJ	RC3H1
	RIF1	RNF168	RNF8	SAMSN1	SANBR	SASH3	SFRP1	SH3KBP1	SHB	SHLD1	SHLD2	SHLD3	SKAP2	SLAMF8	SLC15A4
	SLC25A5	SLC39A10	SLC39A7	SP3	SPI1	ST3GAL1	STAT5B	STAT6	SUPT6H	SWAP70	SYK	SYVN1	TBC1D10C	TBX21	TCF3
	PAXIP1	PCID2	PELI1	PHB1	PHB2	PHF14	PIK3CD	PIK3R1	PIK3R2	PIK3R3	PKN1	PLCG2	PLCL2	POU2AF1	PPP2R3C
	TCIRG1	TFRC	TGFB1	THEMIS2	THOC1	TICAM1	TIRAP	TLR4	TLR9	TNFAIP3	TNFRSF13B	TNFRSF13C	TNFRSF21	TNFRSF4	TNFSF13
	TNFSF13B	TNFSF4	TNIP2	TOP2B	TP53	TP53BP1	TPD52	TRAF3IP2	TXLNA	TYROBP	UNG	VAV3	VCAM1	WNT3A	XBP1
	YY1	ZAP70	ZBTB1	ZBTB7A	ZFP36L1	ZFP36L2									
GOBP B CELL ACTIVATION INVOLVED IN IMMUNE RESPONSE	ABL1	ADA	AICDA	APLF	ATAD5	BATF	BCL3	BCL6	C17orf99	CCR6	CD180	CD19	CD28	CD40	CD40LG
	CDH17	CLCF1	CR1	DLL1	DOCK10	DOCK11	ERCC1	EXO1	EXOSC3	EXOSC6	FCGR2B	FOXP3	GAPT	GPR183	HMCES
	HSPD1	IFNB1	IL10	IL2	IL21	IL27RA	IL4	IL6	IRF8	ITFG2	ITM2A	KMT5B	KMT5C	LFNG	LGALS1
	LIG4	MAD2L2	MFNG	MLH1	MSH2	MSH6	NBN	NDFIP1	NFKBIZ	NKX2-3	NOTCH2	NSD2	PARP3	PAXIP1	PHF14
	PLCG2	PLCL2	POU2AF1	PTK2B	PTPRC	RAG2	RIF1	RNF168	RNF8	SANBR	SHLD1	SHLD2	SHLD3	SLC15A4	SPI1
	ST3GAL1	STAT6	SUPT6H	SWAP70	TBX21	TFRC	TGFB1	THOC1	TLR4	TNFSF13	TNFSF4	TP53BP1	UNG	XBP1	
GOBP B CELL APOPTOTIC PROCESS	ADA	AURKB	BAK1	BAX	BCL10	BCL2	BCL6	BTK	CD74	CRKL	FNIP1	FOXP1	HSH2D	IL10	IL2
	IRS2	LYN	MIF	MIR17HG	NFKBIZ	NOC2L	ORMDL3	PDCD1	PKN1	SLC39A1(	TNFRSF21	TRAF3IP2			
GOBP B CELL CHEMOTAXIS	CH25H	CXCL13	CYP7B1	GAS6	HSD3B7	PIK3CD	PTK2B	XCL1							
GOBP B CELL DIFFERENTIATION	ABL1	ADA	ADAM17	ADGRG3	AICDA	AQP8	ATM	BAD	BAK1	BAX	BCL2	BCL3	BCL6	BLNK	BTK
	C17orf99	CARD11	CD19	CD27	CD40LG	CD79A	CD79B	CDH17	CEBPG	CLCF1	CMTM7	CR1	CR2	DCAF1	DCLRE1C
	DLL1	DNAJB9	DOCK10	DOCK11	EP300	EZH2	FCGR2B	FCRL3	FLT3	FNIP1	FZD9	GPR183	GPS2	HDAC4	HDAC5
	HDAC9	HHEX	HMGB3	ID2	IFNA1	IFNA10	IFNA13	IFNA14	IFNA16	IFNA17	IFNA2	IFNA21	IFNA4	IFNA5	IFNA6
	IFNA7	IFNA8	IFNB1	IFNE	IFNK	IFNW1	IKZF3	IL10	IL11	IL2	IL21	IL2RG	IL4	IL4I1	IL6
	IL7	IL9	INHA	INHBA	INPP5D	IRF2BP2	IRF8	ITFG2	ITGA4	ITGB1	ITM2A	JAK3	KIT	KLF6	LFNG
	LGALS1	LRRC8A	LYL1	MALT1	MFNG	MIR17HG	MMP14	MS4A1	MSH2	NCKAP1L	NFAM1	NFKBIZ	NHEJ1	NKX2-3	NOTCH2
	NTRK1	ONECUT1	PAX5	PCID2	PHF14	PIK3CD	PIK3R1	PIK3R2	PIK3R3	PLCG2	PLCL2	POU2AF1	PPP2R3C	PRKDC	PTK2B
	PTPN2	PTPN6	PTPRC	PTPRJ	RABL3	RAG1	RAG2	RBPJ	SFRP1	SLAMF8	SLC25A5	SLC39A7	SP3	SPI1	ST3GAL1
	STAT5B	SYK	SYVN1	TCF3	TCIRG1	TLR9	TNFSF13B	TOP2B	TP53	TPD52	TRAF3IP2	VCAM1	XBP1	YY1	ZBTB1
	ZBTB7A	ZFP36L1	ZFP36L2												
GOBP B CELL LINEAGE COMMITMENT	BCL2	PRKDC	RAG2	SLAMF8	TCF3	TP53									
GOBP B CELL HOMEOSTASIS	ABL1	ADA	BAK1	BAX	BCL10	BCL2	BCL2L11	CAMLG	CASP3	CD74	DOCK10	DOCK11	FOXP3	GAPT	HIF1A
	IL7R	LYN	MEF2C	MIF	MIR17HG	NCKAP1L	PKN1	PPP2R3C	RC3H1	RC3H2	SASH3	SH2B2	SOS1	SOS2	SPNS2
	TNFAIP3	TNFRSF13B	TNFSF13B	TRAF3IP2											
GOBP B CELL MEDIATED IMMUNITY	AICDA	APLF	ATAD5	B2M	BATF	BCL10	BCL3	BCL6	BTK	C17orf99	C1QA	C1QB	C1QBP	C1QC	C1R
	C1RL	C1S	C2	C3	C4A	C4B	C4BPA	C4BPB	C5	C6	C7	C8A	C8B	C8G	C9
	CARD9	CCR6	CD19	CD226	CD27	CD28	CD40	CD40LG	CD46	CD55	CD70	CD74	CD81	CFI	CLCF1
	CLU	CR1	CR1L	CR2	CSF2RB	ERCC1	EXO1	EXOSC3	EXOSC6	FCER1G	FCER2	FCGR1A	FCGR2B	FCGR3A	FOXJ1
	FOXP3	GAPT	HLA-DMA	HLA-DMB	HLA-DOA	HLA-DOB	HLA-DPA1	HLA-DPB1	HLA-DQA1	HLA-DQA	HLA-DQB1	HLA-DQB2	HLA-DRA	HLA-DRB1	HLA-DRB3
	HLA-DRB4	HLA-DRB5	HLA-E	HLA-G	HMCES	HPX	HSPD1	IGHG1	IL10	IL13RA2	IL2	IL21R	IL27RA	IL2RB	IL4
	IL9	IL9R	INPP5D	IRF7	KMT5B	KMT5C	LIG4	LTA	MAD2L2	MASP2	MBL2	MLH1	MPL	MSH2	MSH6
	MYD88	NBN	NDFIP1	NECTIN2	NFKBIZ	NOD2	NSD2	PARP3	PAXIP1	PRKCD	PTPN6	PTPRC	RIF1	RNF168	RNF8
	SANBR	SERPING1	SHLD1	SHLD2	SHLD3	SLA2	SLC15A4	STAT6	SUPT6H	SUSD4	SVEP1	SWAP70	TBX21	TCIRG1	TFRC
	TGFB1	THOC1	TLR8	TNF	TNFSF13	TNFSF4	TP53BP1	TRAF3IP2	TREM2	TREX1	UNG	XCL1	ZP3		
GOBP B CELL PROLIFERATION	ABL1	ADA	AHR	ATAD5	ATM	BAX	BCL2	BCL6	BMI1	BST1	BTK	CARD11	CASP3	CD180	CD19
	CD22	CD300A	CD320	CD38	CD40	CD40LG	CD70	CD74	CD79A	CD81	CDKN1A	CHRNB2	CLCF1	CR2	CTLA4
	CTPS1	EPHB2	FCGR2B	FCRL3	GAPT	GPR183	HSPD1	IFNA1	IFNA10	IFNA13	IFNA14	IFNA16	IFNA17	IFNA2	IFNA21
	IFNA4	IFNA5	IFNA6	IFNA7	IFNA8	IFNB1	IFNE	IFNK	IFNW1	IKZF3	IL10	IL13	IL2	IL21	IL4
	IL5	IL7	IL7R	IL9	INPP5D	IRS2	LEF1	LYN	MEF2C	MIF	MIR185	MNDA	MS4A1	MZB1	NCKAP1L
	NFATC2	NFKBIZ	PAWR	PELI1	PKN1	PLCL2	PRKCD	PRLR	PTPRC	RAG2	RASGRP1	RC3H1	SASH3	SHB	SLC39A10
	TFRC	TICAM1	TIRAP	TLR4	TLR9	TNFRSF13B	TNFRSF13C	TNFRSF21	TNFRSF4	TNFSF13B	TYROBP	VAV3	WNT3A		
GOBP B CELL PROLIFERATION INVOLVED IN IMMUNE RESPONSE	ABL1	CD180	CD19	GAPT	PLCL2	TLR4									
GOBP B CELL RECEPTOR SIGNALING PATHWAY	ABL1	BANK1	BAX	BCAR1	BCL2	BLK	BLNK	BMX	BTK	CD19	CD22	CD300A	CD38	CD79A	CD79B
	CD81	CMTM3	CTLA4	FCGR2B	FCRL3	FOXP1	GCSAM	GCSAML	GPS2	GRB2	IGHA1	IGHA2	IGHD	IGHE	IGHG1
	IGHG2	IGHG3	IGHG4	IGHM	IGKC	IGLC1	IGLC3	IGLC6	IGLC7	ITK	KLHL6	LAT2	LCK	LIME1	LPXN
	LYN	MAPK1	MEF2C	MIR18A	MIR19A	MIR34A	MNDA	MS4A1	NCKAP1L	NFAM1	NFATC2	NFKB1	NFKBIA	NFKBIZ	PIK3CD
	PLCG2	PLCL2	PLEKHA1	PRKCB	PRKCH	PTPN22	PTPN6	PTPRC	RFTN1	SH2B2	SLC39A10	SOS1	STAP1	SYK	TEC
	TMIGD2	VAV3													
GOBP GERMINAL CENTER B CELL DIFFERENTIATION	ADA	CDH17	IL21	IL6	IRF8	ITFG2	PHF14	POU2AF1	SPI1						
GOBP GERMINAL CENTER FORMATION	ADA	ADAM17	BCL3	BCL6	CXCL13	FOXJ1	KLHL6	MEF2C	NFKB2	PKN1	RC3H1	TNFAIP3	TNFSF13B	UNC13D	
GOBP IMMATURE B CELL DIFFERENTIATION	ABL1	ATM	FNIP1	IRF2BP2	KIT	LRRC8A	PRKDC	RAG1	RAG2	SPI1	SYVN1	TRAF3IP2			
GOBP INFLAMMATORY RESPONSE TO ANTIGENIC STIMULUS	A2M	BTK	C3	CCR7	CD28	CD6	CD68	CD81	CNR1	CXCR2	CYSLTR1	ELANE	EXT1	FCGR1A	FCGR2B
	FCGR3A	FGR	FURIN	FUT7	FYN	GATA3	GPR17	GPX1	HCK	HLA-DRE	HLA-E	HMGB1	HMGB2	IGHG1	IL10
	IL12B	IL1F10	IL1RN	IL20RB	IL25	IL2RA	IL31RA	IL36A	IL36B	IL36G	IL36RN	IL37	IL5RA	KARS1	LTA
	LYN	MAPK14	MIR105-1	MIR19A	MIR19B1	MIR6869	MKRN2	NLRP6	NOD2	NOTCH1	NOTCH2	NPY	NPY5R	OPRM1	PARK7
	PLA2G2D	PLCG1	PLK2	PNMA1	PSMA1	PSMB4	RAB44	RASGRP1	RBPJ	RHBDF2	SELENOS	SRC	SYK	TNF	TREM2
	TREX1	YES1	ZP3												
GOBP ISOTYPE SWITCHING TO IGA ISOTYPES	CCR6	MLH1	MSH2	NSD2	TGFB1	THOC1	TNFSF13								
GOBP ISOTYPE SWITCHING TO IGG ISOTYPES	ATAD5	CD28	CD40	FOXP3	HSPD1	IL2	IL27RA	IL4	MLH1	MSH2	NDFIP1	PAXIP1	PTPRC	SLC15A4	TBX21
GOBP MARGINAL ZONE B CELL DIFFERENTIATION	BCL3	CDH17	DLL1	DOCK10	DOCK11	LFNG	MFNG	NOTCH2	PTK2B						
GOBP MATURE B CELL DIFFERENTIATION	ADA	BCL3	BCL6	C17orf99	CD19	CDH17	CMTM7	CR1	DLL1	DOCK10	DOCK11	FCGR2B	GPR183	IL10	IL2
	IL21	IL2RG	IL6	IRF8	ITFG2	ITM2A	LFNG	LGALS1	MALT1	MFNG	NFKBIZ	NKX2-3	NOTCH2	PHF14	PLCG2
	PLCL2	POU2AF1	PTK2B	RAG2	SLAMF8	SPI1	ST3GAL1	XBP1							
GOBP MATURE B CELL DIFFERENTIATION INVOLVED IN IMMUNE RESPONSE	ADA	BCL3	BCL6	C17orf99	CDH17	CR1	DLL1	DOCK10	DOCK11	FCGR2B	GPR183	IL10	IL2	IL21	IL6
	IRF8	ITFG2	ITM2A	LFNG	LGALS1	MFNG	NFKBIZ	NKX2-3	NOTCH2	PHF14	PLCG2	POU2AF1	PTK2B	RAG2	SPI1
	ST3GAL1	XBP1													
GOBP NEGATIVE REGULATION OF B CELL ACTIVATION	ATM	BANK1	BCL6	BTK	CASP3	CD300A	CR1	CTLA4	FCGR2B	FOXJ1	FOXP3	HMGB3	ID2	IL10	INHA
	INHBA	INPP5D	LAPTM5	LYN	MIR185	MNDA	NDFIP1	PARP3	PAWR	PKN1	RC3H1	SAMSN1	SFRP1	TBC1D10C	THOC1
	TNFAIP3	TNFRSF13B	TNFRSF21	TYROBP											
GOBP NEGATIVE REGULATION OF B CELL APOPTOTIC PROCESS	ADA	AURKB	BCL10	BCL2	BCL6	CD74	FOXP1	HSH2D	IL2	IRS2	MIF	MIR17HG	NOC2L	ORMDL3	PDCD1
	SLC39A10														
GOBP NEGATIVE REGULATION OF B CELL DIFFERENTIATION	BCL6	CR1	HMGB3	ID2	INHA	INHBA	SFRP1								
GOBP NEGATIVE REGULATION OF B CELL MEDIATED IMMUNITY	BCL6	C4BPA	C4BPB	CD46	CR1	CR1L	CR2	FCGR2B	FOXJ1	FOXP3	NDFIP1	PARP3	PTPN6	SUSD4	SVEP1
	THOC1														
GOBP NEGATIVE REGULATION OF B CELL PROLIFERATION	ATM	BTK	CASP3	CD300A	CTLA4	FCGR2B	IL10	INPP5D	LYN	MIR185	MNDA	PAWR	PKN1	RC3H1	TNFRSF13B
	TNFRSF21	TYROBP													
GOBP NEGATIVE REGULATION OF B CELL RECEPTOR SIGNALING PATHWAY	CD22	CD300A	FCGR2B	FCRL3	GPS2	LPXN	MIR34A	PLCL2							
GOBP PLASMA CELL DIFFERENTIATION	BCL6	CR1	IL10	IL2	ITM2A	LGALS1	NFKBIZ	NKX2-3	XBP1						
GOBP POSITIVE REGULATION OF B CELL ACTIVATION	ADA	AKIRIN2	ATAD5	BAD	BCL2	BCL6	BMI1	BST1	BTK	CARD11	CD27	CD28	CD320	CD38	CD40
	CD74	CD81	CDKN1A	CHRNB2	CLCF1	EPHB2	EXOSC3	EXOSC6	FCRL3	GPR183	HMCES	IL10	IL13	IL2	IL21
	IL2RG	IL4	IL5	IL6	IL7	INPP5D	IRS2	KMT5B	KMT5C	MAD2L2	MEF2C	MIF	MLH1	MMP14	MSH2
	NCKAP1L	NFATC2	NOD2	NSD2	PAXIP1	PCID2	PELI1	PPP2R3C	PRLR	PTPRC	RIF1	SASH3	SH3KBP1	SHLD1	SHLD2
	SHLD3	SLC39A10	SPI1	STAT5B	STAT6	SYK	TBX21	TFRC	TGFB1	TICAM1	TIRAP	TLR4	TLR9	TNFRSF13C	TNFRSF4
	TNFSF13	TNFSF13B	TNFSF4	TNIP2	TP53BP1	VAV3	WNT3A	XBP1							
GOBP POSITIVE REGULATION OF B CELL DIFFERENTIATION	BAD	BTK	CD27	IL10	IL2	IL2RG	IL7	INPP5D	MMP14	NCKAP1L	PCID2	PPP2R3C	SPI1	STAT5B	SYK
	XBP1														
GOBP POSITIVE REGULATION OF B CELL MEDIATED IMMUNITY	ATAD5	BTK	C17orf99	C3	CD226	CD28	CD40	CLCF1	EXOSC3	EXOSC6	FCER2	FCGR1A	HLA-E	HMCES	HPX
	IL2	IL4	KMT5B	KMT5C	LTA	MAD2L2	MLH1	MSH2	NECTIN2	NOD2	NSD2	PAXIP1	PTPRC	RIF1	SHLD1
	SHLD2	SHLD3	STAT6	TBX21	TFRC	TGFB1	TNF	TNFSF13	TNFSF4	TP53BP1	TREM2	XCL1			
GOBP POSITIVE REGULATION OF B CELL PROLIFERATION	ADA	ATAD5	BCL2	BCL6	BMI1	BST1	BTK	CARD11	CD320	CD38	CD40	CD74	CD81	CDKN1A	CHRNB2
	CLCF1	EPHB2	FCRL3	GPR183	IL13	IL2	IL21	IL4	IL5	IL7	IRS2	MEF2C	MIF	NCKAP1L	NFATC2
	PELI1	PRLR	PTPRC	SASH3	SLC39A10	TFRC	TICAM1	TIRAP	TLR4	TLR9	TNFRSF13C	TNFRSF4	TNFSF13B	VAV3	WNT3A
GOBP POSITIVE REGULATION OF B CELL RECEPTOR SIGNALING PATHWAY	CD81	CMTM3	FOXP1	MIR18A	MIR19A	NFAM1	PRKCB	PRKCH	SLC39A10	STAP1					
GOBP PRO B CELL DIFFERENTIATION	FLCN	FLT3	FNIP1	HES1	HES5	LIG4	NOTCH1	NUDT21	PRKDC	SOS1	SOS2	SOX4			
GOBP REGULATION OF B CELL ACTIVATION	ADA	AHR	AKIRIN2	APLF	ATAD5	ATM	BAD	BANK1	BCL2	BCL6	BMI1	BST1	BTK	CARD11	CASP3
	CD19	CD22	CD27	CD28	CD300A	CD320	CD38	CD40	CD74	CD81	CDKN1A	CHRNB2	CLCF1	CR1	CTLA4
	EPHB2	EXOSC3	EXOSC6	FCGR2B	FCRL3	FOXJ1	FOXP3	GPR183	HMCES	HMGB3	ID2	IKZF3	IL10	IL13	IL2
	IL21	IL27RA	IL2RG	IL4	IL4I1	IL5	IL6	IL7	INHA	INHBA	INPP5D	IRS2	KMT5B	KMT5C	LAPTM5
	LYN	MAD2L2	MEF2C	MIF	MIR17HG	MIR185	MLH1	MMP14	MNDA	MSH2	MZB1	NCKAP1L	NDFIP1	NFAM1	NFATC2
	NOD2	NSD2	PARP3	PAWR	PAXIP1	PCID2	PELI1	PKN1	PPP2R3C	PRLR	PTPN6	PTPRC	RC3H1	RIF1	SAMSN1
	SASH3	SFRP1	SH3KBP1	SHLD1	SHLD2	SHLD3	SLAMF8	SLC15A4	SLC39A10	SPI1	STAT5B	STAT6	SUPT6H	SYK	TBC1D10C
	TBX21	TFRC	TGFB1	THEMIS2	THOC1	TICAM1	TIRAP	TLR4	TLR9	TNFAIP3	TNFRSF13B	TNFRSF13C	TNFRSF21	TNFRSF4	TNFSF13
	TNFSF13B	TNFSF4	TNIP2	TP53BP1	TYROBP	VAV3	WNT3A	XBP1	ZFP36L1	ZFP36L2					
GOBP REGULATION OF B CELL APOPTOTIC PROCESS	ADA	AURKB	BAX	BCL10	BCL2	BCL6	BTK	CD74	FNIP1	FOXP1	HSH2D	IL10	IL2	IRS2	LYN
	MIR17HG	NOC2L	ORMDL3	PDCD1	SLC39A10	MIF									
GOBP REGULATION OF B CELL DIFFERENTIATION	BAD	BCL6	BTK	CARD11	CD27	CR1	FCRL3	HMGB3	ID2	IKZF3	IL10	IL2	IL2RG	IL4I1	IL7
	INHA	INHBA	INPP5D	MIR17HG	MMP14	NCKAP1L	NFAM1	PCID2	PPP2R3C	PTPN6	SFRP1	SLAMF8	SPI1	STAT5B	SYK
	TLR9	XBP1	ZFP36L1	ZFP36L2											
GOBP REGULATION OF B CELL MEDIATED IMMUNITY	APLF	ATAD5	BCL6	BTK	C17orf99	C3	C4BPA	C4BPB	CD226	CD28	CD40	CD46	CLCF1	CR1	CR1L
	CR2	EXOSC3	EXOSC6	FCER2	FCGR1A	FCGR2B	FOXJ1	FOXP3	HLA-E	HMCES	HPX	IL10	IL2	IL27RA	IL4
	KMT5B	KMT5C	LTA	MAD2L2	MLH1	MSH2	NDFIP1	NECTIN2	NOD2	NSD2	PARP3	PAXIP1	PTPN6	PTPRC	RIF1
	SHLD1	SHLD2	SHLD3	SLC15A4	STAT6	SUPT6H	SUSD4	SVEP1	TBX21	TFRC	TGFB1	THOC1	TNF	TNFSF13	TNFSF4
	TP53BP1	TREM2	XCL1												
GOBP REGULATION OF B CELL PROLIFERATION	ADA	AHR	ATAD5	ATM	BCL2	BCL6	BMI1	BST1	BTK	CARD11	CASP3	CD22	CD300A	CD320	CD38
	CD40	CD74	CD81	CDKN1A	CHRNB2	CLCF1	CTLA4	EPHB2	FCGR2B	FCRL3	GPR183	IKZF3	IL10	IL13	IL2
	IL21	IL4	IL5	IL7	INPP5D	IRS2	LYN	MEF2C	MIF	MIR185	MNDA	MZB1	NCKAP1L	NFATC2	PAWR
	PELI1	PKN1	PRLR	PTPRC	RC3H1	SASH3	SLC39A10	TFRC	TICAM1	TIRAP	TLR4	TLR9	TNFRSF13B	TNFRSF13C	TNFRSF21
	TNFRSF4	TNFSF13B	TYROBP	VAV3	WNT3A										
GOBP REGULATION OF B CELL RECEPTOR SIGNALING PATHWAY	CD19	CD22	CD300A	CD81	CMTM3	FCGR2B	FCRL3	FOXP1	GCSAM	GCSAML	GPS2	LPXN	LYN	MIR18A	MIR19A
	MIR34A	NFAM1	PLCL2	PRKCB	PRKCH	PTPN22	SLC39A10	STAP1							
GOBP REGULATION OF TYPE B PANCREATIC CELL DEVELOPMENT	AKT1	BAD	BMAL1	CLOCK	GSK3A	GSK3B	NKX6-1	RFX3	RHEB						
GOBP TOLERANCE INDUCTION DEPENDENT UPON IMMUNE RESPONSE	AIRE	CD274	FOXP3	HAVCR2	HLA-G	PSG9									
GOCC B CELL RECEPTOR COMPLEX	CD79A	CD79B	LIME1	SYK	TMIGD2										
HP ABNORMAL B CELL MORPHOLOGY	ADA	ALG12	ARHGEF1	ATP11A	B2M	BACH2	BTK	CARD11	CASP10	CD19	CD79B	CTNNBL1	CTPS1	DCLRE1B	DCLRE1C

	DOCK8	ELF4	FAS	FASLG	FCHO1	FNIP1	GFI1	HYOU1	ICOS	IGHM	IGLL1	IKBKB	IKBKG	IKZF1	IKZF3
	IL21	IL2RA	IL6R	IRF2BP2	IVNS1ABP	JAK3	KNSTRN	LCP2	LRBA	MCM10	MYD88	MYSM1	NBN	NCKAP1L	NFKB2
	NHEJ1	PIK3CD	PIK3R1	PLCG2	POMP	PRIM1	PRKCD	PTPRC	RAC2	RAG1	RAG2	RASGRP1	REL	RIPK1	SASH3
	SLC39A7	SOCS1	SP110	SPI1	STAT1	SYK	TBX2	TCF3	TET2	TLR8	TOM1	TRNT1	UNC119	WDR1	
HP ABNORMAL B CELL SUBSET DISTRIBUTION	ARHGEF1	BACH2	CD19	CTNNBL1	CTPS1	ELF4	FNIP1	HYOU1	ICOS	IKBKB	IL21	IL6R	IRF2BP2	LCP2	LRBA
	PIK3CD	PIK3R1	PLCG2	POMP	PRKCD	RASGRP1	REL	SOCS1	SP110	TET2	TOM1				
HP ABNORMAL PROPORTION OF CLASS SWITCHED MEMORY B CELLS	CD19	CTNNBL1	FNIP1	HYOU1	ICOS	IL21	IL6R	IRF2BP2	LCP2	LRBA	PIK3CD	PLCG2	PRKCD	REL	SOCS1
	TET2														
HP ABNORMALITY OF HUMORAL IMMUNITY	ADA	ADA2	ADAM17	ADAMTS3	AICDA	AK2	ALB	ALG12	ARHGEF1	ATM	ATP6AP1	ATP6AP2	B2M	BACH2	BCL10
	BLM	BLNK	BTK	C1QA	C1QB	C1QC	C1S	C3	C4A	C4B	C5	C6	C7	C8A	C8B
	C9	CARD10	CARD11	CARD9	CARMIL2	CARS1	CASP10	CASP8	CAVIN1	CCBE1	CCND1	CD19	CD247	CD27	CD28
	CD3D	CD3E	CD3G	CD40	CD40LG	CD46	CD55	CD70	CD79A	CD79B	CD81	CDCA7	CDSN	CFB	CFD
	CFH	CFHR1	CFHR3	CFI	CFP	CIITA	CISD2	CNBP	COG6	COL1A1	COL7A1	CR2	CSNK2A1	CTLA4	CTNNBL1
	CTPS1	CXCR4	DCLRE1C	DNASE1L3	DNMT3B	DOCK8	ELANE	EPG5	ERCC2	ERCC3	EXTL3	FAS	FASLG	FAT4	FLNA
	FNIP1	FOXP3	GALK1	GBA1	GPC3	GPC4	GPR35	GTF2E2	GTF2H5	HELLS	HLA-DQA1	HLA-DQB1	HLA-DRB1	HYOU1	ICOS
	IFIH1	IFNGR1	IGHG2	IGHM	IGKC	IGLL1	IKBKB	IKBKG	IKZF1	IL12A	IL12RB1	IL21	IL21R	IL2RA	IL2RB
	IL2RG	IL4R	IL6R	IL6ST	IL7R	IPO8	IRAK1	IRAK4	IRF2BP2	IRF5	ITCH	ITK	IVNS1ABP	JAK3	KLHDC8B
	KNSTRN	KRAS	KRT1	KRT14	KRT5	KRT74	KRT9	LAMTOR2	LAT	LIG1	LIG4	LMNA	LMNB2	LRBA	LRRC8A
	MAGT1	MAN2B1	MASP2	MGAT2	MMEL1	MOGS	MPLKIP	MS4A1	MS4A2	MSN	MST1	MTHFD1	MTOR	MUC5B	MVK
	MYD88	MYSM1	NBN	NCKAP1L	NFE2L2	NFKB1	NFKB2	NHEJ1	NHLRC2	NLRP1	NRAS	NSMCE3	NTRK1	OAS1	OTULIN
	PACS1	PEPD	PGM3	PIGT	PIK3CD	PIK3CG	PIK3R1	PLA2G7	PLCG2	PLVAP	PMM2	POLE	POMP	POU2AF1	PRF1
	PRIM1	PRKCD	PSMB4	PSMB8	PSMB9	PSTPIP1	PTEN	PTPRC	RAC2	RAG1	RAG2	RASGRP1	RBM8A	REL	RFX5
	RFXANK	RFXAP	RIPK1	RMRP	RNF113A	RNF168	RNU4ATAC	RPA1	RTEL1	SAMD9L	SASH3	SCARB2	SEMA4D	SERPING1	SETX
	SFTPA2	SFTPC	SH2D1A	SH3KBP1	SIK3	SKIC2	SKIC3	SLC25A13	SLC35C1	SLC39A7	SLC46A1	SLC5A6	SLC7A7	SMARCAL1	SON
	SP110	SPI1	SPIB	SPINK5	SPP1	SPPL2A	STAT2	STAT3	STAT4	STAT5B	STING1	STX11	STXBP2	SYK	TARS1
	TCF3	TCF4	TCN2	TERT	TFRC	TGFB1	TIMM8A	TLR7	TLR8	TNFRSF1	TNFRSF13B	TNFRSF13C	TNFRSF1B	TNFSF12	TNFSF15
	TNPO3	TOM1	TONSL	TP53	TPP2	TRMU	TRNT1	TTC7A	TYK2	TYMS	UNC13D	UNG	VPS33A	VPS45	WAS
	XIAP	ZAP70	ZBTB24	ZEB2	ZNF341										
HP ABSENT CIRCULATING B CELLS	BTK	FNIP1	IGLL1	SLC39A7	SPI1										
HP ABSENCE OF LYMPH NODE GERMINAL CENTER	ADA	CD40	CD40LG	EPHB4	PTPRC	SP110									
HP INCREASED B CELL COUNT	CARD11	CASP10	FAS	FASLG	NCKAP1L	PRKCD	PTPRC	RASGRP1							

Supplementary Table S4: The mean result of 10 single runs of the machine learning (ML) model for the training set in RA vs. HC.

ML model	AUC(95%CI)	cutoff(95%CI)	Accuracy(95%CI)	Sensitivity(95%CI)	Specificity(95%CI)	Positive predictive value(95%CI)	Negative predictive value(95%CI)	F1 score(95%CI)	Kappa(95%CI)
XGBoost	0.999 (0.997-1.000)	0.906(0.877-0.934)	0.975(0.969-0.981)	0.979(0.972-0.985)	1.000(1.000-1.000)	1.000(1.000-1.000)	0.864(0.837-0.892)	0.989(0.986-0.993)	0.912(0.892-0.931)
logistic	0.988 (0.972-1.000)	0.664(0.616-0.712)	0.963(0.955-0.972)	0.972(0.962-0.982)	0.959(0.949-0.969)	0.992(0.991-0.994)	0.833(0.807-0.858)	0.982(0.977-0.987)	0.869(0.848-0.890)
LightGBM	1.000 (NaN-NaN)	0.873(0.832-0.915)	0.993(0.993-0.993)	1.000(1.000-1.000)	1.000(1.000-1.000)	1.000(1.000-1.000)	0.958(0.956-0.960)	1.000(1.000-1.000)	0.974(0.973-0.975)
RandomForest	1.000 (NaN-NaN)	0.700(0.631-0.769)	0.988(0.983-0.992)	1.000(1.000-1.000)	1.000(1.000-1.000)	1.000(1.000-1.000)	0.926(0.897-0.954)	1.000(1.000-1.000)	0.953(0.935-0.972)
AdaBoost	1.000 (NaN-NaN)	0.695(0.660-0.730)	0.992(0.990-0.993)	1.000(1.000-1.000)	1.000(1.000-1.000)	1.000(1.000-1.000)	0.949(0.937-0.961)	1.000(1.000-1.000)	0.969(0.961-0.976)
CNB	0.744 (0.667-0.821)	0.747(0.682-0.812)	0.676(0.655-0.698)	0.629(0.599-0.660)	0.969(0.942-0.995)	0.992(0.985-0.999)	0.326(0.313-0.339)	0.769(0.748-0.790)	0.328(0.311-0.345)
MLP	0.816 (0.781-0.849)	0.915(0.773-1.057)	0.790(0.617-0.963)	0.764(0.554-0.975)	0.968(0.949-0.988)	NaN(NaN-NaN)	0.585(0.435-0.736)	NaN(NaN-NaN)	0.607(0.412-0.802)
SVM	0.980 (0.952-1.000)	0.610(0.569-0.651)	0.962(0.958-0.965)	0.970(0.965-0.975)	0.961(0.952-0.970)	0.992(0.991-0.994)	0.829(0.812-0.847)	0.981(0.979-0.983)	0.867(0.857-0.877)
KNN	0.992 (0.982-1.000)	0.840(0.762-0.918)	0.691(0.460-0.922)	0.947(0.933-0.962)	0.975(0.961-0.988)	NaN(NaN-NaN)	0.524(0.359-0.689)	NaN(NaN-NaN)	0.533(0.303-0.763)

Supplementary Table S5: The mean result of 10 single runs of the machine learning (ML) model for the testing set in RA vs. HC.

ML model	AUC(95%CI)	cutoff(95%CI)	Accuracy(95%CI)	Sensitivity(95%CI)	Specificity(95%CI)	Positive predictive value(95%CI)	Negative predictive value(95%CI)	F1 score(95%CI)	Kappa(95%CI)
XGBoost	0.972 (NaN-NaN)	0.906(0.877-0.934)	0.950(0.934-0.966)	0.974(0.958-0.990)	0.975(0.926-1.024)	0.993(0.985-1.002)	0.754(0.663-0.845)	0.983(0.974-0.993)	0.807(0.732-0.882)
logistic	0.989 (NaN-NaN)	0.664(0.616-0.712)	0.958(0.942-0.975)	0.972(0.953-0.992)	0.978(0.934-1.021)	0.983(0.967-0.998)	0.851(0.768-0.934)	0.977(0.964-0.990)	0.856(0.797-0.916)
LightGBM	0.966 (NaN-NaN)	0.873(0.832-0.915)	0.925(0.896-0.954)	0.925(0.884-0.966)	0.975(0.942-1.008)	0.973(0.956-0.989)	0.737(0.619-0.854)	0.948(0.920-0.976)	0.722(0.632-0.811)
RandomForest	0.979 (NaN-NaN)	0.700(0.631-0.769)	0.922(0.883-0.961)	0.967(0.939-0.996)	0.980(0.941-1.019)	0.983(0.969-0.998)	0.729(0.589-0.869)	0.975(0.954-0.996)	0.743(0.617-0.869)
AdaBoost	0.983 (NaN-NaN)	0.695(0.660-0.730)	0.897(0.862-0.933)	0.952(0.904-1.001)	1.000(1.000-1.000)	0.989(0.974-1.004)	0.600(0.478-0.723)	0.970(0.937-1.002)	0.662(0.549-0.774)
CNB	0.755 (0.601-0.908)	0.747(0.682-0.812)	0.678(0.624-0.731)	0.667(0.600-0.734)	1.000(1.000-1.000)	0.972(0.952-0.993)	0.302(0.255-0.349)	0.787(0.739-0.835)	0.293(0.231-0.356)
MLP	0.808 (NaN-NaN)	0.915(0.773-1.057)	0.786(0.590-0.982)	0.766(0.540-0.992)	0.971(0.934-1.009)	NaN(NaN-NaN)	0.662(0.467-0.857)	NaN(NaN-NaN)	0.615(0.399-0.831)
SVM	0.983 (NaN-NaN)	0.610(0.569-0.651)	0.956(0.941-0.970)	0.974(0.957-0.990)	0.983(0.951-1.016)	0.987(0.976-0.997)	0.799(0.729-0.868)	0.980(0.969-0.991)	0.825(0.787-0.864)
KNN	0.974 (NaN-NaN)	0.840(0.762-0.918)	0.694(0.471-0.917)	0.968(0.949-0.987)	0.969(0.928-1.010)	NaN(NaN-NaN)	0.492(0.319-0.664)	NaN(NaN-NaN)	0.489(0.266-0.712)

Supplementary Table S6: The mean result of 10 single runs of the machine learning (ML) model for the training set in RA vs. OA.

ML model	AUC(95%CI)	cutoff(95%CI)	Accuracy(95%CI)	Sensitivity(95%CI)	Specificity(95%CI)	Positive predictive value(95%CI)	Negative predictive value(95%CI)	F1 score(95%CI)	Kappa(95%CI)
XGBoost	1.000 (NaN-NaN)	0.606(0.549-0.664)	0.993(0.993-0.993)	1.000(1.000-1.000)	1.000(1.000-1.000)	1.000(1.000-1.000)	0.946(0.943-0.949)	1.000(1.000-1.000)	0.968(0.966-0.970)
logistic	0.921 (0.862-0.981)	0.810(0.786-0.835)	0.855(0.842-0.868)	0.865(0.848-0.882)	0.843(0.817-0.869)	0.974(0.970-0.978)	0.466(0.430-0.503)	0.916(0.907-0.924)	0.519(0.485-0.552)
LightGBM	1.000 (NaN-NaN)	0.751(0.693-0.809)	0.993(0.993-0.993)	1.000(1.000-1.000)	1.000(1.000-1.000)	1.000(1.000-1.000)	0.946(0.942-0.950)	1.000(1.000-1.000)	0.968(0.966-0.970)
RandomForest	1.000 (NaN-NaN)	0.675(0.616-0.734)	0.987(0.979-0.995)	1.000(1.000-1.000)	1.000(1.000-1.000)	1.000(1.000-1.000)	0.913(0.866-0.959)	1.000(1.000-1.000)	0.945(0.913-0.978)
AdaBoost	1.000 (NaN-NaN)	0.541(0.535-0.547)	0.993(0.993-0.993)	1.000(1.000-1.000)	1.000(1.000-1.000)	1.000(1.000-1.000)	0.946(0.943-0.950)	1.000(1.000-1.000)	0.968(0.967-0.970)
CNB	0.532 (0.430-0.634)	0.605(0.532-0.679)	0.473(0.438-0.507)	0.425(0.381-0.468)	0.867(0.825-0.909)	0.957(0.943-0.971)	0.176(0.165-0.188)	0.585(0.541-0.628)	0.106(0.095-0.117)
MLP	0.618 (0.533-0.703)	1.048(0.901-1.195)	0.533(0.349-0.717)	0.487(0.271-0.704)	0.904(0.841-0.968)	NaN(NaN-NaN)	0.251(0.184-0.318)	NaN(NaN-NaN)	0.221(0.114-0.327)
SVM	0.813 (0.690-0.936)	0.883(0.873-0.893)	0.851(0.844-0.858)	0.881(0.875-0.887)	0.702(0.677-0.727)	0.952(0.947-0.958)	0.446(0.432-0.461)	0.915(0.911-0.920)	0.461(0.442-0.480)
KNN	0.939 (0.897-0.980)	0.980(0.941-1.019)	0.191(0.066-0.315)	0.735(0.690-0.781)	0.983(0.951-1.016)	NaN(NaN-NaN)	0.150(0.105-0.194)	NaN(NaN-NaN)	0.041(-0.039-0.121)

Supplementary Table S7: The mean result of 10 single runs of the machine learning (ML) model for the testing set in RA vs. OA.

分类模型	AUC(95%CI)	cutoff(95%CI)	准确度(95%CI)	灵敏度(95%CI)	特异度(95%CI)	阳性预测值(95%CI)	阴性预测值(95%CI)	F1分数(95%CI)	Kappa(95%CI)
XGBoost	0.801 (NaN-NaN)	0.606(0.549-0.664)	0.891(0.858-0.925)	0.901(0.844-0.958)	0.801(0.704-0.897)	0.936(0.909-0.963)	NaN(NaN-NaN)	0.917(0.878-0.956)	0.444(0.251-0.636)
logistic	0.935 (0.844-0.998)	0.810(0.786-0.835)	0.840(0.799-0.881)	0.839(0.754-0.924)	1.000(1.000-1.000)	0.978(0.962-0.994)	0.427(0.327-0.528)	0.898(0.835-0.961)	0.474(0.376-0.572)
LightGBM	0.869 (NaN-NaN)	0.751(0.693-0.809)	0.906(0.891-0.920)	0.924(0.889-0.959)	0.874(0.781-0.967)	0.958(0.936-0.979)	0.619(0.471-0.767)	0.939(0.920-0.958)	0.568(0.474-0.661)
RandomForest	0.798 (NaN-NaN)	0.675(0.616-0.734)	0.880(0.831-0.929)	0.808(0.624-0.993)	0.849(0.740-0.959)	0.940(0.911-0.968)	0.540(0.357-0.723)	0.825(0.641-1.010)	0.482(0.291-0.673)
AdaBoost	0.895 (0.748-1.000)	0.541(0.535-0.547)	0.883(0.850-0.916)	0.890(0.812-0.967)	0.878(0.787-0.968)	0.952(0.931-0.973)	0.505(0.404-0.607)	0.915(0.867-0.964)	0.497(0.375-0.618)
CNB	0.482 (NaN-NaN)	0.605(0.532-0.679)	0.406(0.364-0.447)	0.379(0.293-0.465)	0.969(0.926-1.012)	0.907(0.848-0.965)	0.139(0.101-0.176)	0.525(0.436-0.615)	0.022(-0.018-0.061)
MLP	0.611 (NaN-NaN)	1.048(0.901-1.195)	0.534(0.367-0.701)	0.561(0.306-0.816)	0.910(0.791-1.029)	NaN(NaN-NaN)	0.224(0.174-0.274)	NaN(NaN-NaN)	0.166(0.073-0.258)
SVM	0.721 (0.384-0.966)	0.883(0.873-0.893)	0.800(0.772-0.828)	0.874(0.737-1.011)	0.680(0.521-0.839)	0.933(0.906-0.960)	0.330(0.245-0.415)	0.887(0.790-0.984)	0.294(0.198-0.390)
KNN	0.769 (NaN-NaN)	0.980(0.941-1.019)	0.183(0.064-0.302)	0.823(0.764-0.882)	0.707(0.583-0.831)	NaN(NaN-NaN)	0.131(0.100-0.163)	NaN(NaN-NaN)	0.015(-0.014-0.043)
